# The steroid hormone ADIOL promotes learning by reducing neural kynurenic acid levels

**DOI:** 10.1101/gad.350745.123

**Published:** 2023

**Authors:** George A. Lemieux, Shinja Yoo, Lin Lin, Mihir Vohra, Kaveh Ashrafi

**Affiliations:** Department of Physiology, University of California, San Francisco, San Francisco, California 94143, USA

**Keywords:** 5-androstenediol, estrogen receptor β, kynurenic acid, kynurenine pathway, nuclear hormone receptor

## Abstract

Reductions in brain kynurenic acid levels, a neuroinhibitory metabolite, improve cognitive function in diverse organisms. Thus, modulation of kynurenic acid levels is thought to have therapeutic potential in a range of brain disorders. Here we report that the steroid 5-androstene 3β, 17β-diol (ADIOL) reduces kynurenic acid levels and promotes associative learning in *Caenorhabditis elegans*. We identify the molecular mechanisms through which ADIOL links peripheral metabolic pathways to neural mechanisms of learning capacity. Moreover, we show that in aged animals, which normally experience rapid cognitive decline, ADIOL improves learning capacity. The molecular mechanisms that underlie the biosynthesis of ADIOL as well as those through which it promotes kynurenic acid reduction are conserved in mammals. Thus, rather than a minor intermediate in the production of sex steroids, ADIOL is an endogenous hormone that potently regulates learning capacity by causing reductions in neural kynurenic acid levels.

## Abstract (precis)

In this study, Lemieux et al. show that the small molecule F17 promotes feeding and cognitive function in *C. elegans* by engaging the intestinal receptor NHR-131 to increase synthesis of the steroid hormone 5-androstene 3β, 17β-diol (ADIOL). ADIOL subsequently stimulates neuronal NHR-91, reducing levels of the neuroinhibitory metabolite kynurenic acid, which enhances learning capacity in young and aged animals.

Kynurenine pathway metabolites are derived from tryptophan catabolism and function as signaling molecules by acting through specific receptors in multiple organ systems (Cervenka et al. 2017; Kennedy et al. 2017; Schwarcz and Stone 2017). For example, kynurenic acid (KYNA) is an antagonist of glutamatergic receptors, including *N*-methyl *D*-aspartate receptors (NMDARs) (Stone 1993), and an agonist of GPR-35, a G-protein-coupled receptor (Wang et al. 2006). Kynurenine pathway metabolites are well suited as signaling molecules because their levels vary in response to multiple physiological perturbations, including caloric restriction, hypoxia, exercise, infection, and inflammation (Agudelo et al. 2014, 2018; Lemieux et al. 2015; Olenchock et al. 2016; Vohra et al. 2017; Sorgdrager et al. 2019; Lerch et al. 2022; Wyant et al. 2022). Consistent with its actions as a competitive antagonist of NMDARs, reduced KYNA promotes learning capacity in mice and rats, while elevated KYNA has detrimental effects (Chess and Bucci 2006; Chess et al. 2007; Potter et al. 2010; Pocivavsek et al. 2011; Alexander et al. 2012; Kozak et al. 2014). Increased KYNA characterizes several human brain diseases, including neurodegenerative diseases, major depression, and schizophrenia (Connick et al. 1989; Schwarcz et al. 2001; Kepplinger et al. 2005; Nilsson et al. 2005; Olsson et al. 2010; González-Sánchez et al. 2020). Attenuation of KYNA levels is considered a potential therapeutic approach in some of these disease conditions (Stone and Darlington 2013; Savitz 2020).

*Caenorhabditis elegans* exhibits associative learning and memory (Sasakura and Mori 2013). A commonly used learning paradigm uses the odor of butanone as a conditioning stimulus to which *C. elegans* can form either attractive or aversive associations, depending on the specific training regimen used (Colbert and Bargmann 1995; Torayama et al. 2007; Kauffman et al. 2010). The molecular mechanisms that underlie formation of these associations include proteins such as the glutamatergic AMPA and NMDA receptors, which also function in mammalian learning paradigms (Kauffman et al. 2010; Peng et al. 2011; Vohra et al. 2017). Reduced KYNA levels enhance NMDAR-dependent forms of associative learning and memory, while increased KYNA levels have detrimental effects in *C. elegans* (Vohra et al. 2017, 2018; Lin et al. 2020). These effects are due to KYNA's inhibition of the activity of RIM neurons, a pair of NMDAR-expressing neurons (Vohra et al. 2017, 2018). These neurons also serve as a key site of KYNA production for the control of both feeding and learning behaviors (Lemieux et al. 2015; Vohra et al. 2017; Lin et al. 2020). We previously observed that in *C. elegans* the beneficial effects of caloric/dietary restriction on learning capacity are accounted for by reductions in KYNA (Vohra et al. 2017), and KYNA accumulation accounts for a significant decrease in the learning capacity of aged *C. elegans* (Vohra et al. 2018). Thus, in *C. elegans* and mammals, reduced levels of KYNA improve learning and memory, and KYNA levels increase with age (Moroni et al. 1988; Gramsbergen et al. 1992; Kepplinger et al. 2005).

Given that KYNA accumulates with age and in several disease-associated states and negatively impacts learning in animals, it has been of great interest to elucidate the molecular signaling mechanisms that control KYNA levels in the nervous system. Here, we identify an endogenous signaling cascade that links a peripheral transcriptional pathway to neural KYNA levels. An unexpected finding emerging from our work is that increases in 5-androstene 3β, 17β-diol (ADIOL) potently reduce kynurenic acid levels to promote both feeding and learning capacity. We show that ADIOL functions as a learning-promoting agent even in aged animals that exhibit deficits in learning capacity. The physiological functions of ADIOL, which is also found in mammals, including humans, have thus far been poorly understood. At best, ADIOL has been considered a minor intermediate in the steroidogenic pathways of the adrenal glands or the gonads (Adams 1985). Our work demonstrates that rather than a mere intermediate, ADIOL is an endogenous hormone that potently regulates learning capacity.

## Results

### F17 reduces KYNA levels, increasing the pharyngeal pumping rate and learning capacity

**Figure 1.**
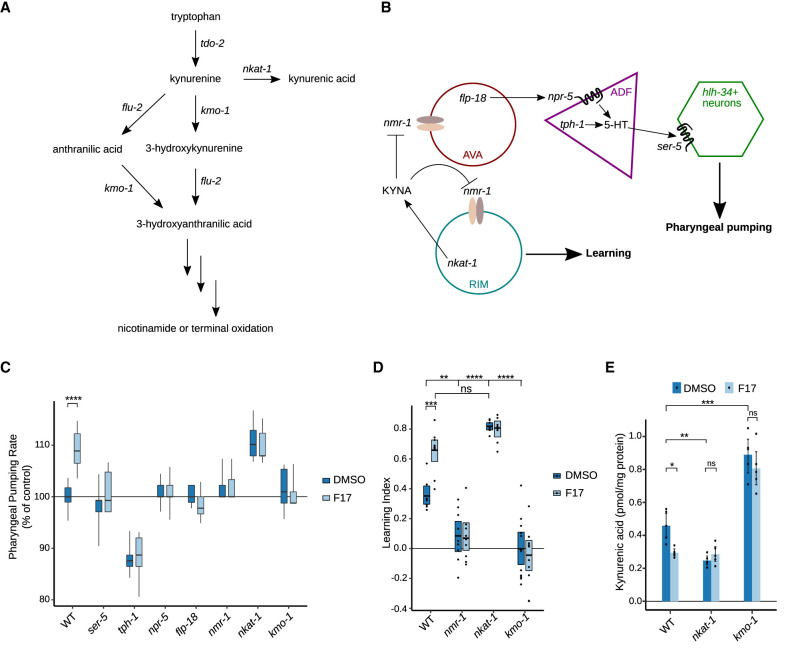
F17 lowers KYNA levels and promotes short-term associative learning. (*A*) Relationship of kynurenine pathway metabolites to pathway enzymes. (*B*) Anatomic and genetic organization of mechanisms linking KYNA levels to learning capacity and pharyngeal pumping. While KYNA levels affect both pharyngeal pumping and learning capacity through modulation of NMDARs, these behavioral outcomes are mediated by distinct NMDAR-expressing neurons. RIM-, AVA-, ADF-, and *hlh-34*-expressing neurons indicate neurons previously identified as sites of actions of the indicated genes. *nmr-1*, *flp-18*, *npr-5*, *tph-1*, and *ser-5* encode for an NMDAR subunit, a neuropeptide Y-like ligand, a neuropeptide Y-like receptor, tryptophan hydroxylase (the rate-limiting enzyme in serotonin biosynthesis), and a serotonergic G-protein-coupled receptor, respectively. (*C*) Effects of vehicle control (DMSO) or F17 on pharyngeal pumping rates in wild type, KYNA-deficient *nkat-1* mutants, high-KYNA-containing *kmo-1* mutants, and a set of mutants previously shown to block the elevated pharyngeal pumping rates of *nkat-1* animals. *N* = 12–63 animals per condition. Statistical evaluation by ANOVA (Holm-corrected). (*D*) Associative learning in wild type (WT) and the indicated mutants treated with either DMSO as a vehicle control or F17. Statistical evaluation by ANOVA (Tukey's HSD). *n* = 7–12 assays per condition. (*E*) KYNA metabolite levels in wild-type animals and *nkat-1* and *kmo-1* mutants treated with either DMSO as vehicle control or F17. *n* = 5 independent biological samples per condition. Statistical evaluation by ANOVA (Holm's correction). In *C*–*E*, *P*-values are as follows: (*) *P* < 0.05, (**) *P* < 0.01, (***) *P* < 0.001, (****) *P* < 0.0001, (ns) *P* > 0.05.

KYNA is synthesized by the oxidation of kynurenine, a reaction catalyzed by kynurenine aminotransferases (Fig. 1A; Stone and Darlington 2013). The *C. elegans nkat-1* gene encodes for a kynurenine aminotransferase (Yu et al. 2006; Lemieux et al. 2015). We previously demonstrated that *nkat-1* mutants are deficient in KYNA (Lemieux et al. 2015). *C. elegans* modulate their food intake rate by increasing or decreasing pharyngeal pumping rates, the mechanism by which they ingest food (Lemieux and Ashrafi 2015). KYNA-deficient *nkat-1* mutants exhibit constitutively elevated pharyngeal pumping rates even under ad libitum feeding conditions (Lemieux et al. 2015). This is because reductions in neural KYNA levels are a mechanism through which *C. elegans* assess the experience of fasting to subsequently increase their food intake rate upon encountering a food source postfast (Avery and Horvitz 1990; Lemieux et al. 2015). The pumping regulatory effects of KYNA are dependent on NMDAR activity in specific neurons and a peptidergic signaling cascade that is initiated from these neurons (Fig. 1B; Lemieux et al. 2015). As already indicated, KYNA-deficient *nkat-1* mutants also exhibit elevated learning capacity by modulating the activity of specific NMDAR-expressing neurons (Vohra et al. 2017). Components of NMDAR signaling as well as *nkat-1* are expressed in only a few neurons (Lemieux et al. 2015; Vohra et al. 2017). Previous work established that production of KYNA from the RIM neurons is sufficient for animals to have wild-type patterns of pumping and learning capacity under the conditions tested (Lemieux et al. 2015; Vohra et al. 2017). While KYNA production from the RIM neurons regulates both pumping rate and learning capacity, these behaviors are regulated by NMDAR signaling in distinct neurons (Fig. 1B; Lemieux et al. 2015; Vohra et al. 2017). As described below, we leveraged the knowledge that KYNA levels affect pumping rate to facilitate identification of KYNA-dependent learning regulatory mechanisms.

In addition to elevated pumping rate and enhanced learning capacity compared with wild-type animals (Lemieux et al. 2015; Vohra et al. 2017), *nkat-1* mutants also exhibit reduced levels of Nile Red, a dye used to identify metabolic perturbations in *C. elegans* (Ashrafi et al. 2003). The mechanistic basis of reduced Nile Red content in *nkat-1* mutants is unknown. A previous unrelated phenotypic screen of several thousand small molecules in *C. elegans* identified 4-hydroxy-1-(2-methylpropyl)-*N*-(5-methyl-1,3-thiazol-2-yl)-2-oxoquinoline-3-carboxamide (F17) (Lemieux et al. 2011), at the time a biologically uncharacterized small molecule that elicited the same high pharyngeal pumping and low Nile Red phenotypes exhibited by *nkat-1* mutants. Although the original identification of F17 was completely unconnected to the kynurenine pathway, we wondered whether the phenotypes elicited by F17 may be due to reductions in KYNA. We found that elevation of pharyngeal pumping in response to overnight treatment with F17 requires the very same molecular components as those that underlie the *nkat1* mutant’s elevated rate (Fig. 1B). These include the NMDAR encoded by *nmr-1*, a neuropeptide Y-like ligand (FLP-18), and its receptor (NPR-5), as well as *tph-1* and *ser-5*, which encode for a key serotonin biosynthetic enzyme and a serotonergic receptor, respectively (Fig. 1C). The already elevated pharyngeal pumping rate of *nkat-1* mutants was not further increased by F17 (Fig. 1C).

While reductions in KYNA promote learning and pharyngeal pumping, these behaviors are not simply consequences of each other, since KYNA exerts its effects on these two behavioral processes by acting on NMDARs of different neurons (Lemieux et al. 2015; Vohra et al. 2017). Therefore, we directly tested whether F17 treatment also affects learning capacity. Overnight treatment with F17 robustly improved short-term associative learning with butanone (referred to here as learning) in wild-type animals (Fig. 1D). Further mimicking the effects of *nkat-1* mutants, the enhanced learning capacity of F17-treated animals was dependent on NMDARs, and treatment of *nkat-1* mutants with F17 did not enhance the already elevated learning capacity of these animals (Fig. 1D). Next, we directly measured KYNA from whole-animal extracts and found that animals treated with F17 overnight exhibited reduced KYNA levels relative to vehicle-treated wild-type animals (Fig. 1E). The extent of KYNA reduction in F17-treated animals was similar to that observed previously in *nkat-1* mutants (Lemieux et al. 2015; Vohra et al. 2017), and F17 did not further enhance kynurenic acid reduction in that genetic background (Fig. 1E).

**Figure 2.**
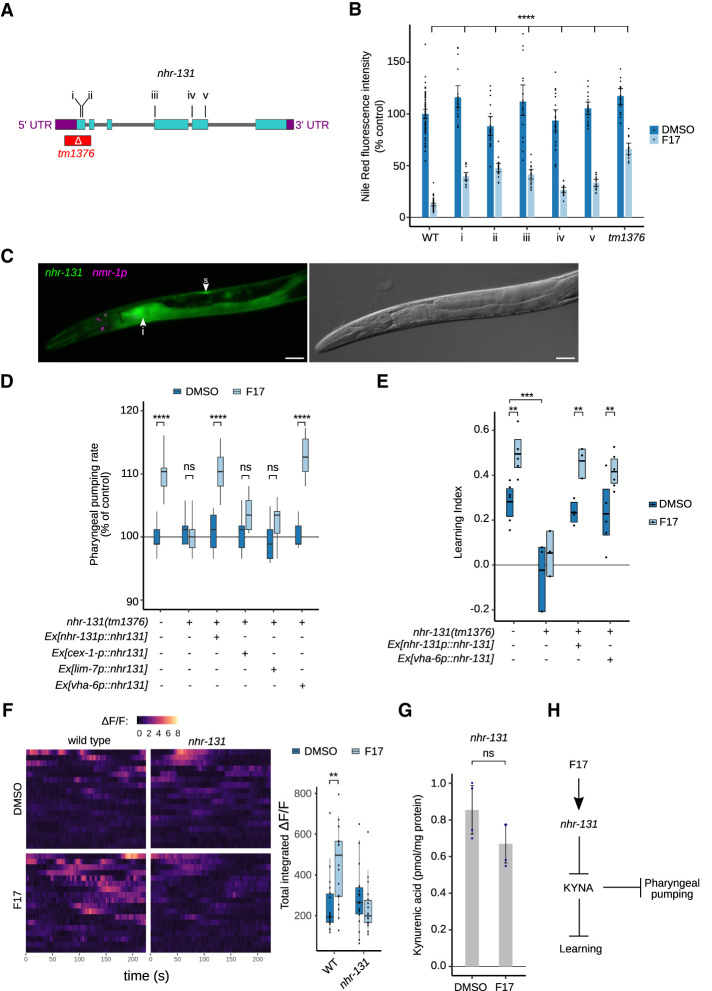
The nuclear hormone receptor NHR-131 is required for F17's biological effects. (*A*) Genomic location of the five identified EMS alleles (locations i–v) and a known deletion allele (*tm1376*) in *nhr-131*. The predicted functional consequence for each allele is missense Gly19Arg (location i), missense Val24Met (location ii), splice-accepting exon 3 (location iii), splice-accepting exon 4 (location iv), and premature stop Trp261STOP (location v). (*B*) Quantification of Nile Red staining in wild type, isolated EMS mutants (locations i–v), and the *nhr-131(tm1376)* deletion mutant. Error bars represent the 95% confidence interval of the mean. Statistical evaluation by ANOVA (Holm's correction). *n* = 12–47 animals per condition. (*C*, *left* panel) Composite fluorescence micrographs of transgenic animals expressing the transcriptional reporters for *nhr-131p::nhr-131bcsGFP* and a coinjection marker (*nmr-1p::mCherry*). Arrowheads indicate the intestine (i) and the somatic gonad (s). (*Right* panel) DIC micrograph of the same animal. Scale bars, 30 μm. (*D*) Pharyngeal pumping rates of L4 stage wild-type, *nhr-131(tm1376)* mutant, and transgenic strains bearing extrachromosomal arrays expressing the *nhr-131* coding sequence using the 5′ upstream sequences from *nhr-131* (used in *C*), *cex-1*, *lim-7*, and *vha-6*. Statistical evaluation by two-way ANOVA (Holm's correction). *n* = 16 animals per condition. (*E*) Associative learning in wild type, *nhr-131* mutants, and *nhr-131* mutants in which a wild-type *nhr-131* transgene was driven by either the *nhr-131* promoter used in *C* or the intestine-specific *vha-6* promoter. Animals were treated with F17 or DMSO alone. *N* = 3–6 trials per condition. Statistical evaluation by ANOVA (Holm's correction). (*F*) Spontaneous Ca^2+^ transients recorded from RIM neurons expressing GCaMP in wild-type and *nhr-131* mutant backgrounds treated with F17 or DMSO alone. Eighteen recordings of the change in fluorescence (Δ*F/F*) over 225 sec per recording are shown. The summed intensity of each recording is plotted. Statistical evaluation by Kruskal–Wallis (Dunn's test). (*G*) KYNA metabolite levels in *nhr-131* mutants treated with F17 or DMSO alone. Statistical evaluation by Welch's *t*-test. *n* = 4 independent biological samples per condition. In *B* and *D*–*G*, *P*-values are as follows: (*) *P* < 0.05, (**) *P* < 0.01, (***) *P* < 0.001, (****) *P* < 0.0001, (ns) *P* > 0.1. (*H*) Inferred pharmaco–genetic–metabolic relationship of F17, *nhr-131*, and KYNA levels and behavior.

Kynurenine aminotransferases and kynurenine mono-oxygenases use the same substrate, kynurenine—the latter enzyme irreversibly oxidizing kynurenine to 3-hydroxy kynurenine (Fig. 1A; Stone and Darlington 2013). *C. elegans* mutant in *kmo-1*, encoding a kynurenine mono-oxygenase, have higher levels of kynurenine and KYNA than wild-type animals (van der Goot et al. 2012; Lemieux et al. 2015), exhibit diminished short-term associative learning, and do not transiently hyperelevate their pharyngeal pumping rates after fasting (Lemieux et al. 2015; Vohra et al. 2017). In contrast to wild-type animals, the pharyngeal pumping rate and defective learning ability of *kmo-1* mutants were unresponsive to overnight F17 treatment (Fig. 1C,D). In *kmo-1* mutant animals, the elevated levels of kynurenic acid were not significantly reduced by treatment overnight with F17 (Fig. 1E), suggesting that elevated KYNA levels interfere with the behavioral effects of F17. However, the ability of *kmo-1* mutants to block F17 was dependent on the exposure time to F17. Extending the exposure time from overnight (16–20 h) (Fig. 1C–E) to 2 d (40–44 h) (Supplemental Fig. S1A) rendered *kmo-1* mutants susceptible to F17's pumping stimulatory effects. Thus, while F17's behavioral effects are sensitive to kynurenic acid levels, these behavioral effects do not, in an absolute manner, depend on *kmo-1* function. Together, our findings indicate that F17 treatment reduces KYNA and recapitulates the Nile Red, pharyngeal pumping, and learning phenotypes of KYNA-deficient *nkat-1* mutants, but these effects of F17 are blocked when KYNA is elevated to supraphysiological levels.

### nhr-131, a peripheral nuclear hormone receptor required for learning

To identify mechanisms of action of F17 and infer possible targets, the chemical similarity between F17 and all other chemicals with known protein targets was queried systematically (Lemieux et al. 2013). However, in this instance, the computational search was unfruitful. The sensitivity of F17's behavioral phenotype to KYNA levels suggested possible transcriptional regulation of kynurenine pathway genes by F17, but qPCR analysis of the expression of several pathway genes including *nkat-1* and *kmo-1* failed to provide evidence of such a regulatory mechanism (Supplemental Fig. S1B). Therefore, we turned to an unbiased forward random mutagenesis approach to identify novel genetic perturbations that block or suppress the effects of F17. As indicated, similar to *nkat-1* mutants, F17 treatment reduces Nile Red staining. We exploited this property of F17 in a visual screen for viable, fertile F2 mutants that were resistant to F17's ability to depress Nile Red staining (Supplemental Fig. S2A). We surmised that mutants identified in such a screen may shed light on the mechanisms by which F17 treatment causes reductions in KYNA. After screening 24,000 EMS mutagenized genomes; backcrossing suppressing strains to the nonmutagenized, wild-type genetic background; and deep sequencing, we identified five strains containing different, independent point mutations in the gene *nhr-131*, which is predicted to encode a previously uncharacterized nuclear hormone receptor (Fig. 2A). All the mutations appeared to be loss-of-function modifications to the gene because they suppressed F17's Nile Red reduction to an extent similar to that of a strain containing a deletion allele, *nhr-131(tm1376)*, obtained from the National Bioresource Center of Japan (Fig. 2A,B). The *nhr-131(tm1376)* allele was used for all subsequent experiments. To explore the anatomical location where *nhr-131* is expressed, we created bicistronic GFP fusions to the *nhr-131* gene. The transgene was strongly expressed in the intestine and somatic gonad, somewhat weaker in the pharyngeal muscle and a few neural cells, and weakly in the epidermis (Fig. 2C; Supplemental Fig. S2B), a pattern consistent with that previously observed for this gene based on single-cell RNA profiling (Cao et al. 2017).

We next characterized the *nhr-131* mutant animals in the context of F17-stimulated behaviors. Unlike *kmo-1* mutants, feeding stimulated by extended treatment with F17 was blocked by *nhr-131* mutants (Fig. 2D). Expression of *nhr-131* using the same promoter used in Figure 2C was sufficient to restore the feeding stimulatory activity of F17 to *nhr-131* mutants (Fig. 2D). Transgenic expression of *nhr-131* using a *lim-7* promoter to target the somatic gonad (Voutev et al. 2009) or a *cex-1* promoter to target the RIM neurons (Piggott et al. 2011), a critical center of KYNA synthesis, failed to restore F17 responsiveness to *nhr-131* mutants (Fig. 2D). In contrast, reconstitution of *nhr-131* in the intestinal cells of *nhr-131* mutants using the *vha-6* promoter (Oka et al. 2001) restored the ability of *nhr-131* mutants to elevate their feeding rates upon F17 treatment (Fig. 2D).

We also found that *nhr-131* mutants are deficient in learning relative to control-treated wild-type animals and are unresponsive to F17 (Fig. 2E). The learning deficiency of *nhr-131* and other mutants with basally defective learning described above (*nmr-1* and *kmo-1*) cannot simply be attributed to general defects in movement (Supplemental Fig. S2C), alterations in naive chemotaxis to butanone (Supplemental Fig. S2D), or general inability to be attracted to odors sensed by AWC sensory neurons (Supplemental Fig. S2E). Given its prominent expression and sufficiency for feeding behavior stimulated by F17, we examined whether NHR-131 activity in intestinal cells is sufficient for wild-type learning capacity. In *nhr-131* mutants with ectopically expressed *nhr-131*, both basal short-term learning ability and F17-induced enhanced learning were restored whether *nhr-131* was driven using its putative endogenous promoter or an intestine-specific promoter (Fig. 2E). The combined data suggest that F17 promotes its behavioral phenotypes through engagement of *nhr-131* in the intestine.

We previously established that the NMDAR-expressing RIM interneurons are among the few *C. elegans* neurons that can generate KYNA as they express *nkat-1* (Lemieux et al. 2015). Moreover, we demonstrated that learning regulatory effects of KYNA require NMDAR function in the RIM neurons and that the learning-enhancing effects of KYNA reduction result in enhanced Ca^2+^ activity in the RIM neurons (Vohra et al. 2017, 2018; Lin et al. 2020). Therefore, we measured the effect of F17 and *nhr-131* loss on spontaneous Ca^2+^ activity in RIM neurons using a GCaMP transgene in animals conditioned with butanone. Upon F17 treatment, the frequency and total integrated intensity of Ca^2+^ transients were significantly elevated in wild-type animals (similar to those previously reported for *nkat-1* mutants) (Vohra et al. 2017) but not in *nhr-131* mutants (Fig. 2F). Additionally, loss of *nhr-131* largely abrogated the KYNA-reducing effects of F17 (Fig. 2G). These results indicated that F17 is dependent on *nhr-131* to reduce KYNA levels and enhance the activity of RIM neurons, previously shown to be critical in NMDAR-dependent learning paradigms (Fig. 2H). However, it is important to note the *nhr-131* mutants exhibit learning deficits even in the absence of F17 treatment. Thus, while loss of *nhr-131* blocks the KYNA-reducing effects of F17, it is likely that *nhr-131* mutants also affect learning capacity through KYNA-independent mechanisms.

### Functional examination of NHR-131 transcriptional targets

**Figure 3.**
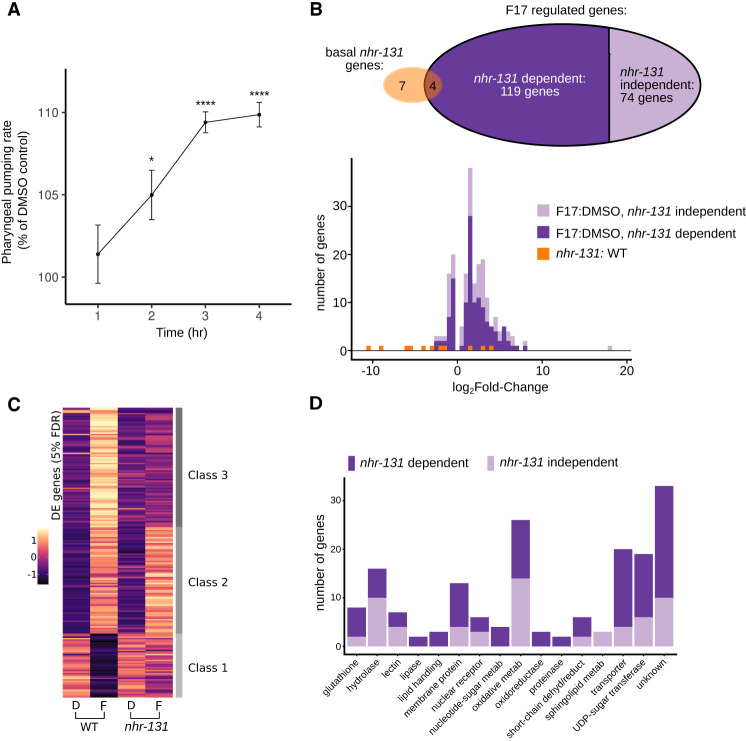
Elucidation of a transcriptional program regulated by F17 and *nhr-131*. (*A*) Pharyngeal pumping rates after 1, 2, 3, and 4 h of exposure to F17 normalized to those of animals exposed to DMSO vehicle for the same time period. The mean (points) and standard error of the mean (error bars) for determinations from 19–20 animals per condition are plotted. Statistical evaluation by Welch's *t*-test comparing F17 treatment versus DMSO alone at each time point is denoted. (*) *P* < 0.05, (****) *P* < 0.0001. (*B*) Relationship of genes induced by F17 to *nhr-131*’s activity. The Venn diagram shows the number of genes regulated by F17 and the subset dependent on *nhr-131.* The histogram shows the distribution of the magnitude of gene expression changes at 5% FDR for each of the indicated contrasts. In F17-treated versus DMSO-treated animals, the fraction of genes in each bin whose change in expression is sensitive to *nhr-131* is indicated. (*C*) Mean row-normalized gene expression data for all significant F17- and *nhr-131*-regulated genes. Wild type (WT) and *nhr-131* mutants were treated with either F17 (*F*) or DMSO alone (*D*). Rows are ordered by *k*-means clustering into three broad classes. (DE) Differentially expressed, (FDR) false discovery rate. (*D*) The number of genes in each observed molecular function class. In *B* and *D*, F17-induced changes were considered *nhr-131*-dependent if the difference in F17-induced change in wild-type versus *nhr-131* mutants resulted in a Wald test *P*-value of <0.05. All gene identities and expression changes used in *B*–*D* are detailed in Supplemental Data Set S1.

Our findings raised the question of how the activity of NHR-131 in the intestinal cells is sufficient to affect KYNA-regulated neural responses. Since *nhr-131* encodes for an uncharacterized transcription factor, we next sought to identify potential F17-regulated transcriptional targets of NHR-131. We measured relative mRNA differences between both wild type and *nhr-131* mutants exposed to a vehicle control and F17 for 4 h, the exposure time required for F17 to elicit its maximal effects on pharyngeal pumping in wild-type animals (Fig. 3A). Overall, we measured the changes in the expression of 15,413 genes. In wild-type animals, at a 5% false discovery rate, F17 treatment up-regulated 150 and down-regulated 43 gene expressions (Fig. 3B; Supplemental Data Set S1). Sixty-one percent (119 out of 193) of the F17-regulated genes were dependent on *nhr-131* for their expression changes. Without F17 treatment, only eight genes were down-regulated and three were up-regulated when comparing expressions in vehicle-treated *nhr-131* mutants versus wild-type animals (Fig. 3B; Supplemental Data Set S1). Among the F17-regulated genes, we identified three broad classes (Fig. 3C). Class 1 genes were repressed by F17 treatment in wild-type animals but far less so in *nhr-131* mutants. Class 2 genes were stimulated by F17 treatment in wild-type animals and *nhr-131* mutants. Class 3 consisted of genes whose expression was stimulated by F17 treatment dependent on *nhr-131*.

**Figure 4.**
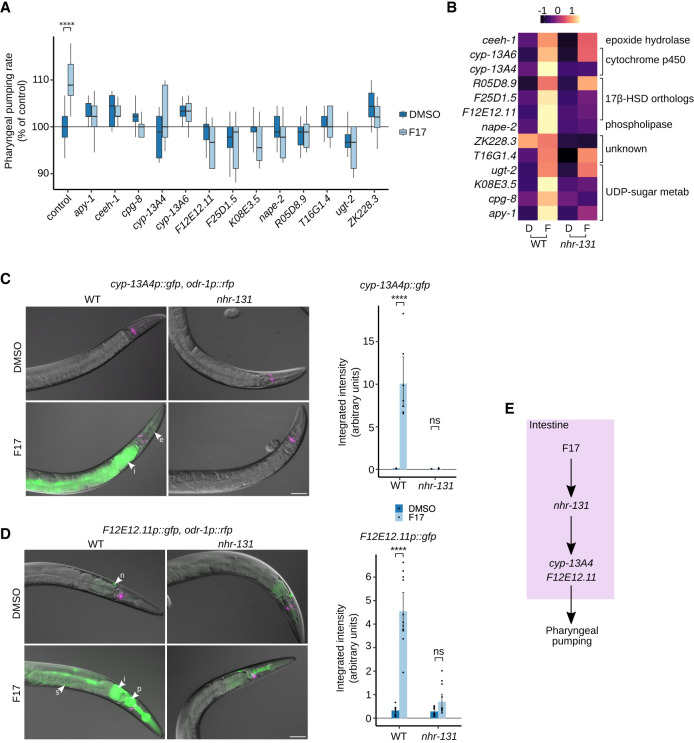
Identification of F17-regulated genes that are required for elevated ad libitum pharyngeal pumping rates. (*A*) RNA inhibitors that block the F17-induced increase in pharyngeal pumping rate without significantly altering the baseline levels of pumping (DMSO controls). While F17 treatment resulted in a significant increase ([****] *P* < 0.0001) in pumping rates of vector control animals, this increase was abrogated for the RNAi clones shown. Compared with F17-treated vector control, all gene inactivations shown resulted in a significant difference (*P* < 0.05) in the pumping rate of F17-treated animals. Statistical evaluation by ANOVA (Holm's correction). *n* = 10–130 per group. (*B*) Mean-normalized gene expression data by row for genes in *A* from wild type (WT) and *nhr-131* mutants treated with DMSO (*D*) or F17 (*F*). (*C*,*D*) Superimposed epifluorescence and DIC micrographs of transgenic animals in both wild-type (WT) and *nhr-131* mutant backgrounds, treated with DMSO alone or F17. *odr-1p::rfp* was used as the coinjection marker. Arrowheads indicate the intestine (i), epidermis (e), neuron (n), pharyngeal muscle (p), and somatic gonad (s). Scale bars, 30 μm. Quantifications for F17-induced transgene up-regulations are shown. *C* shows quantification of GFP fluorescence in the intestine for *n* = 7 animals per condition, and *D* shows quantification in the intestine and pharynx for *n* = 10–11 animals per condition. Statistical evaluation by ANOVA (Holm's correction) is indicated. (****) *P* < 0.0001, (ns) *P* > 0.1. (*E*) Inferred anatomic and pharmacogenetic organization of F17, *nhr-131*, *cyp-13A4*, *F12E12.11*, and pharyngeal pumping.

The functions of F17-regulated genes mostly relate to metabolic roles, including oxidative metabolism, transport, glycosyl transferases, hydrolases, and numerous genes of unknown or unpredictable function (Fig. 3D). F17 did not regulate the expression of *nhr-131*. Also absent from this list are genes encoding enzymes of the kynurenine pathway itself (Supplemental Data Set S1). As the molecular identities of these genes did not reveal obvious connections to the kynurenine pathway, we systematically examined the effect of inactivation of these genes on pharyngeal pumping, an F17-regulated, KYNA-dependent behavior. We chose to measure pharyngeal pumping because this assay has a much higher throughput than learning assays. Using RNAi, we screened through 101 of the F17- or *nhr-131*-regulated genes on pharyngeal pumping. We found that 44 of the attempted gene knockdowns interacted with F17 treatment to alter pumping rates in a variety of manners (Fig. 4A; Supplemental Fig. S3A). Among these, we focused on the 13 RNAi clones that blocked the pumping-increasing effects of F17 without altering basal rates (Fig. 3A), given the unambiguous genetic relationship to F17. Of these 13 gene inactivations, 11 did not affect the elevated pumping phenotype of *nkat-1* mutants (Supplemental Fig. S3B), suggesting that these genes likely function between F17 and KYNA production. The molecular identities of these genes suggested roles in nucleotide sugar biosynthesis and metabolism as well as biosynthesis and metabolism of lipid signaling species including steroids, as discussed below (Fig. 4B). Since we did not verify the extent of gene knockdown for each of the 101 clones tested, we cannot rule out the involvement of genes for which RNAi failed to give us obvious phenotypes. As discussed below, we used mutant strains to follow through on this RNAi screen.

### ADIOL is a KYNA regulatory steroid

Our findings indicate that NHR-131 activity in intestinal cells is sufficient to regulate the KYNA-dependent effects of F17 on pharyngeal pumping and learning. As noted, these behavioral effects depend on neural KYNA production (Lemieux et al. 2015; Vohra et al. 2017; Lin et al. 2020). The predicted molecular identities of several of the genes that blocked the effects of F17 but not the loss of *nkat-1* on pumping raised the possibility that NHR-131 exerts transcriptional control on a steroid biosynthetic pathway, providing a hormonal link between an intestinal transcriptional program and neural KYNA-regulated processes. Production of all steroid hormones starts with the irreversible conversion of cholesterol, a moiety containing 27 carbons, to the 21-carbon steroid pregnenolone (Supplemental Fig. S4; Luu-The 2013). This conversion is mediated by a cytochrome P450 family enzyme known as CYP11A in mammals (Luu-The 2013). Pregnenolone is the common precursor to steroid hormones grouped as mineralocorticoids, glucocorticoids, and sex hormones, including adrenal androgens (Luu-The 2013). The conversion of pregnenolone to any one of its derivatives is determined by the specific enzymes that act in different arms of steroid hormone biosynthesis. For example, the synthesis of steroid hormones containing 19 carbons, collectively known as C19 steroids, depends on another cytochrome P450 family member, CYP17A1 in mammals, and its partner, CYTB5, a cytochrome b5 reductase (Supplemental Fig. S4; Duggal et al. 2018; Kim et al. 2021). The C19 steroids include the well-known sex hormones testosterone and 17β-estradiol, the adrenal androgens dehydroepiandrosterone (DHEA) and androstenedione, and estrone and the poorly understood 5-androstene 3β, 17β-diol (ADIOL) (Supplemental Fig. S4). Importantly, 17β-hydroxysteroid dehydrogenase/reductases (17β-HSDs), which catalyze either the reduction or oxidation of the 17th carbon of the scaffold from 19-carbon steroids (Moeller and Adamski 2009), function in the C19 arm of steroid hormone biosynthesis but not the biosynthesis of 21-carbon-containing glucocorticoids or mineralocorticoids (Supplemental Fig. S4). Among the genes identified in the RNAi screen (Fig. 3A,B), two are predicted to encode 17β-HSDs (*F25D1.5* and *F12E12.11*) along with a pair of cytochrome p450 enzymes (*cyp13A4* and *cyp13A6*). Additionally, another predicted 17β-HSD (*R05D8.9*) and *cytb5.1* (encoding a cytochrome b5 reductase) were among the F17 up-regulated genes (Supplemental Data Set S1). These results suggested that a C19 family steroid may link NHR-131 activity to neural KYNA levels.

To gain insights into anatomical sites of function of the subset of genes potentially involved in steroid biosynthesis, we created transgenic lines expressing GFP fusions to promoter regions of one of the cytochrome P450s (*cyp-13A4*) and one of the putative 17β-HSDs (*F12E12.11*, also known as *nta-1*). Expression of the *cyp-13A4p::gfp* transgene was nearly invisible in wild-type animals but was up-regulated strongly in the intestine and weakly in the epidermis upon F17 treatment, dependent on *nhr-131* (Fig. 4C). We also integrated a fluorescent reporter into the *C. elegans* genome at the *cyp-13A4* locus to generate an endogenously regulated, C-terminally fluorescently tagged translational fusion of this gene. Treatment of these animals with F17 resulted in a strong induction of intestinal fluorescence and hypodermal fluorescence that was identical to that seen using the extrachromosomal promoter fusion reporter for this gene (Supplemental Fig. S3C). Under basal conditions (vehicle control), the 17β-HSD homolog *F12E12.11p::gfp* was expressed in a neural cell in the head and weakly in the intestine and pharyngeal muscle of both wild type and *nhr-131* mutants (Fig. 4D). F17 strongly induced it in the pharyngeal muscle, intestine, and somatic gonad of otherwise wild-type animals. In *nhr-131* mutants, the strong expression in the peripheral tissues upon F17 treatment was significantly attenuated (Fig. 4D).

**Figure 5.**
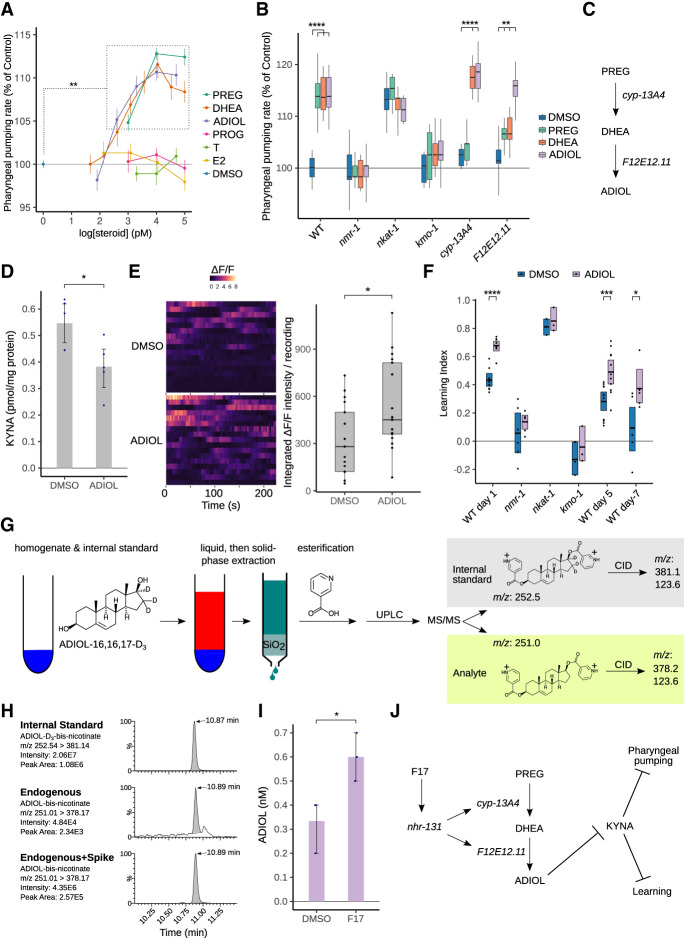
Identification of a steroid biosynthesis pathway that generates ADIOL, which promotes learning in both young and aged adults. (*A*) Dose–response relationship of varying concentrations of pregnenolone (PREG); dehydroepiandrosterone (DHEA); 5-androstene-3β, 17β-diol (ADIOL); progesterone (PROG); testosterone (T); 17β-estradiol (E2); and vehicle (DMSO) on pharyngeal pumping. Points represent the mean and whiskers represent the standard error of the mean of the pumping rates of 12–28 animals per dose. Statistical evaluation by ANOVA (Dunnett's correction). (**) *P* < 0.01 compared with DMSO. (*B*) Pharyngeal pumping rates of wild-type (WT) and mutant animals treated with different steroids or with DMSO alone as a vehicle control. *n* = 15–32 animals per condition. The data were statistically evaluated by ANOVA (Holm's correction). (*C*) Inferred metabolic organization of *cyp-13A4* and *F12E12.11.* (*D*) KYNA levels from wild-type animals treated with ADIOL and DMSO as a control. The same data for DMSO were used in Figure 1C. *n* = 5 independent biological samples per condition. Statistical evaluation by ANOVA (Holm's correction). (*E*) Spontaneous Ca^2+^ transients recorded from RIM neurons expressing GCaMP in animals treated with DMSO or ADIOL and conditioned with butanone prior to imaging. Fifteen recordings of the change in fluorescence (Δ*F/F*) over 225 sec (s) per recording are shown. The summed intensity of each recording is plotted. Statistical evaluation by Wilcoxon rank-sum test. (*F*) Short-term learning performance for wild-type (WT) day 1 animals, kynurenine pathway mutants, and 5-d and 7-d aged animals treated with ADIOL or DMSO alone as a vehicle control. Statistical evaluation by ANOVA (Holm's correction). (*G*) Overview of ADIOL quantification by multiple reaction monitoring LC-MS/MS. Acidified homogenates were spiked with ADIOL-D_3_ as an internal standard and extracted into the nonpolar phase, and the residues from the nonpolar phase were subjected to solid-phase extraction using SiO_2_ cartridges. The residues from the eluates were esterified with nicotinic acid and subjected to UPLC-MS/MS. The mass transitions of doubly protonated ADIOL and ADIOL-D_3_ primary ions involving the loss of nicotinic acid after collision-induced dissociation (CID) were selected for analysis. (*H*) Representative chromatograms of the mass transitions monitored. (*I*) Quantification of endogenous ADIOL from wild-type animals treated with F17 or DMSO alone as a control. *n* = 3 independent biological replicates per condition. Statistical evaluation by two-tailed *t-*test. *P*-values in *A*–*I* are as follows: (*) *P* < 0.05, (***) *P* < 0.001, (****) *P* < 0.0001. (*J*) Pharmacogenetic–metabolic relationship of F17, *nhr-131*, *cyp-13A4*, *F12E12.11*, ADIOL, and KYNA with learning and pharyngeal pumping behaviors.

One interpretation of the above results is that F17 engages an intestine-centered transcriptional program through *nhr-131*, which generates steroid hormones to then affect physiological processes in other tissues (Fig. 4E). Consistent with this, we found that pregnenolone, DHEA, and ADIOL each dose-dependently promoted pharyngeal pumping (Fig. 5A). In contrast, animals treated with testosterone, 17β-estradiol, or progesterone did not exhibit a pumping response. Similar to F17, elevation of pharyngeal pumping by pregnenolone, DHEA, or ADIOL was completely blocked in *nmr-1* and *kmo-1* mutants, and these steroids did not further elevate pumping rates of *nkat-1* mutants (Fig. 5B). Thus, similar to F17 treatment, the pumping effects of these steroids are dependent on NMDAR signaling and blocked by high KYNA levels.

As noted, pregnenolone is a precursor to DHEA, which in turn is a precursor to ADIOL. Stepwise conversion of pregnenolone to DHEA and ADIOL is catalyzed first by CYP17A1 and then 17β-HSDs (Supplemental Fig. S4; Luu-The 2013). Therefore, we tested the possibility that *cyp13A4* and *F12E12.11* may encode for CYP17A- and 17β-HSD-like activities. The pumping-promoting effects of pregnenolone, but not those of DHEA and ADIOL, were abrogated in *cyp13A4* mutants (Fig. 5B). *F12E12.11* mutants exhibited a muted response to both pregnenolone and DHEA but responded to ADIOL (Fig. 5B). Thus, *cyp-13A4* and *F12E12.11* appear to function as part of a steroidogenesis program culminating in ADIOL production (Fig. 5C). Since testosterone and 17β-estradiol can be metabolites of ADIOL yet fail to stimulate feeding, ADIOL rather than testosterone or another downstream metabolite is likely to be the active species. We next used direct measurements to find that ADIOL treatment causes KYNA reduction (Fig. 5D), an effect similar in magnitude to that observed in *nkat-1* mutants (Lemieux et al. 2015) and F17-treated animals.

We next tested the effects of ADIOL on associative learning and its cellular correlates. Similar to F17, ADIOL caused an increase in spontaneous Ca^2+^ activity in RIM neurons compared with vehicle control (Fig. 5E). Wild-type *C. elegans* treated with ADIOL exhibited enhanced learning, comparable with *nkat-1* mutants (Fig. 5F). Similar to F17, in both learning-deficient *nmr-1* and *kmo-1* mutants, ADIOL failed to stimulate learning.

In *C. elegans,* one of the first aging phenotypes is a loss of learning ability such that, by day 7 of adulthood, learning capacity is severely compromised (Kauffman et al. 2010; Vohra et al. 2018). We previously observed that KYNA levels increase with aging in *C*. *elegans* and that reducing KYNA improves learning capacity in aged animals (Vohra et al. 2018). Similarly, learning capacity was markedly improved in day 5 and day 7 adults upon ADIOL treatment, even when the treatment started on day 3 of adulthood (Fig. 4F). To rule out that the learning-promoting effects of ADIOL in aged animals may be due to secondary consequences of this steroid hormone on animal life span, we directly examined the effects of ADIOL on life span. Despite its ability to improve learning capacity in aged animals, ADIOL treatment did not have a beneficial effect on life span (Supplemental Fig. S5A). Thus, ADIOL improves NMDA-dependent learning capacity even in aging animals.

### F17 increases endogenous ADIOL production

The production of ADIOL and almost all other known pregnenolone-derived steroids by *C. elegans* has not been previously characterized. *C. elegans* has been shown to contain pregnenolone and progesterone (Broué et al. 2007), implying that they can catalyze the oxidative cleavage of the isoprenoid hydrocarbon chain of cholesterol. In general, endogenous steroid metabolites are difficult to quantify from tissue matrices owing to their low physiological concentrations and physical–chemical properties that are similar to other more abundant metabolites (Olesti et al. 2021). In order to measure ADIOL from *C. elegans*, we devised an ultraperformance liquid chromatography-mass spectrometry (UPLC-MS/MS) assay (Fig. 5G). Steroids such as 17β-estradiol and ADIOL are especially challenging to analyze by electrospray mass spectrometry due to their poor ionization efficiency and instability during ion flight. Postextraction, pre-LC-MS/MS esterification of such steroids with proton affinity groups improves the electrospray ionization and stabilizes those ions prior to detection (Higashi and Ogawa 2016). We adapted a nicotinoyl esterification method previously used to detect androstane derivatives from human serum (Ke et al. 2019). Endogenous ADIOL was detected as a narrow peak eluting at 10.89 min, and the deuterated ADIOL internal standard eluted at 10.87 min (Fig. 5H). In *C. ele*ga*ns* e*x*tracts spiked with exogenous, unlabeled ADIOL, a single peak was observed that was identical in retention time to the peak in the unspiked extract but larger in area. Thus, ADIOL is an endogenous metabolite of *C. elegans*.

We next measured the effect of F17 treatment on the level of ADIOL produced in *C. elegans*. A standard curve relating peak areas to different concentrations of ADIOL produced a linear response over ADIOL concentrations spanning three orders of magnitude, indicating the quantitative nature of the assay (Supplemental Fig. S5B). We found that F17 treatment significantly increased ADIOL levels in *C. elegans* extracts (Fig. 5I). The estimated EC_50_ for the effect of the addition of exogenous ADIOL on pharyngeal pumping is 320 pM (Supplemental Fig. S5C), indicating that the values obtained from the endogenous measurements are physiologically relevant. We also observed that pregnenolone levels were approximately threefold more abundant than ADIOL in vehicle-treated animals (Supplemental Fig. S5D). F17 did not alter pregnenolone levels in a statistically significant manner, although this may be due to the limited number of independent samples examined here. Thus, the direct biochemical measurements are consistent with the genetic and behavioral results in that F17 treatment increases ADIOL levels, which in turn reduce KYNA levels to promote pharyngeal pumping and learning (Fig. 5J).

### ADIOL functions depend on nhr-91 in RIM neurons

**Figure 6.**
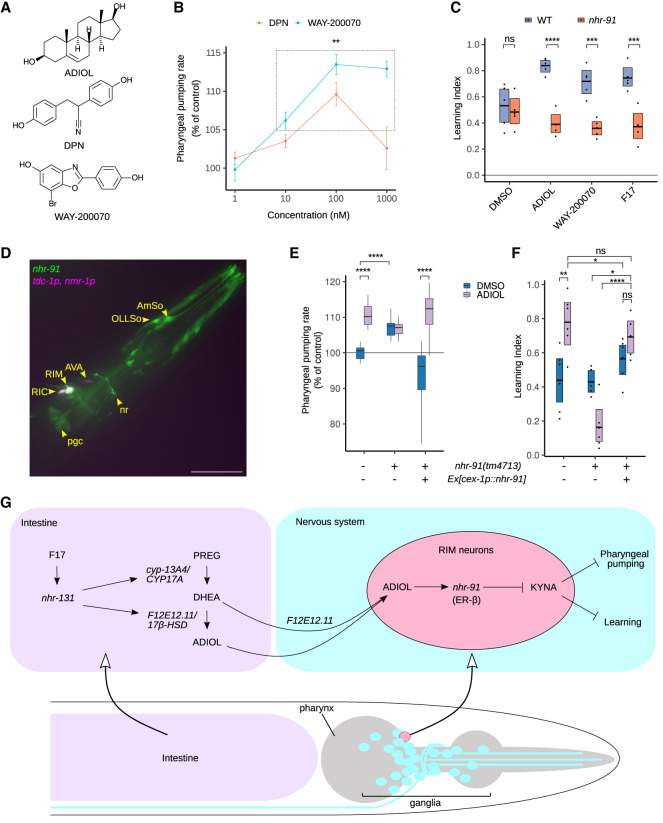
ADIOL functions through *nhr-91* in RIM neurons. (*A*) Chemical structures of ER-β ligands ADIOL, DPN, and WAY-200070. (*B*) Pharyngeal pumping rates of *C. elegans* treated with varying concentrations of WAY-200070 and DPN relative to DMSO-treated control. Points and error bars represent the mean and 95% confidence interval of 10–16 pharyngeal pumping determinations per condition. Points exhibiting means significantly different from DMSO are denoted. (**) *P* < 0.01 ANOVA (Dunnett's test). (*C*) Wild type and *nhr-91* mutants treated with DMSO, ADIOL, WAY-200070, or F17 were assayed for learning ability. *n* = 4–5 trials per condition. (*D*) Maximum intensity *Z*-projection of confocal fluorescence micrographs of the head of a young adult expressing a transgenic array consisting of a bicistronic GFP fusion of the *nhr-91* genomic sequence and transcriptional mCherry fusions under the control of the *tdc-1* and *nmr-1* promoters. Yellow structures represent colocalized mCherry and GFP signals. Arrowheads indicate RIM, RIC, and AVA neurons; nerve ring (nr); pharyngeal gland cell (pgc); and glial socket cells (AmSo and OLLSo). Scale bar, 30 μm. (*E*) Pharyngeal pumping rates of wild type, *nhr-91* mutants, and *nhr-91* mutants ectopically expressing the *nhr-91a* cDNA under the control of the RIM-specific *cex-1* promoter. Animals were treated with ADIOL or DMSO alone as a control. *n* = 31 different animals per condition. (*F*) Associative learning in wild type, nontransgenic *nhr-91* mutants, and *nhr-91* mutants expressing *nhr-91a* cDNA under the control of the RIM-specific *cex-1* promoter. Animals were treated with ADIOL or DMSO alone as a control. *n* = 5–6 independent cohorts per condition. (*G*) Model of the F17/*nhr-131* transcriptional program that regulates ADIOL production to influence behavior in *C. elegans.* F17-induced gene expression up-regulations indicate that biosynthesis of ADIOL occurs in the intestine but do not rule out possible local conversion of DHEA into ADIOL in the nervous system. ADIOL promotes learning and feeding through engaging NHR-91, an ER-β-like nuclear hormone receptor in RIM neurons, to lower KYNA levels. Statistical evaluations are shown in *C*, *E*, and *F*. (***) *P* < 0.001, (****) *P* < 0.0001, (ns) *P* > 0.05 ANOVA (Holm's correction).

While there is a very limited understanding of the physiological effects of ADIOL in any species, it has been characterized as a steroidal ligand for estrogen receptor-β (ER-β), a nuclear hormone receptor (Kuiper et al. 1997) that was discovered based on its sequence homology with ER-α (Kuiper et al. 1996), the key receptor for the estrogen 17β-estradiol. The affinity of the closely related ER-α for ADIOL is about fourfold weaker than that of ER-β (Kuiper et al. 1997). As ER-β has antitumor potential (Mal et al. 2020), there has been interest in developing small molecules that target this receptor. Several pharmacophores, such as diarylpropionitrile (DPN) (Meyers et al. 2001) and WAY-200070 (Malamas et al. 2004), which are structurally distinct from ADIOL (Fig. 6A), more selectively target ER-β over ER-α. We found that treatment with either DPN or WAY-200070 elicited a dose-dependent pumping response reaching the same upper limit as that seen with ADIOL (Fig. 6B). Thus, we surmised that a target pharmacologically similar to ER-β is encoded in the *C. elegans* genome.

Using sequence similarity alone, no single nuclear hormone receptor of *C. elegans* emerges as a clear counterpart of human ER-β. Instead, there are several sequences with modest homology, including within the predicted ligand binding domains of the receptors. To functionally determine whether any receptors might be a target of the ER-β ligands, we used RNAi to systematically examine the 12 *C. elegans* nuclear hormones with the most sequence similarity to human ER-β. Some of these RNAi inactivations altered the baseline pumping rates of animals in the absence of ADIOL, while others had no effects (Supplemental Fig. S6A). Combining ADIOL treatment with RNAi led to changes in pumping rates in all the conditions, in some cases increasing the pumping rate relative to DMSO-treated animals, while in other cases decreasing it. Two exceptions to this pattern were animals subjected to RNAi of either *nhr-91* or *nhr-19*, whose pumping rate did not change in response to ADIOL treatment (Supplemental Fig. S6A). We subsequently used deletion mutants and found that both *nhr-19* and *nhr-91* mutants also block the learning-increasing effects of ADIOL (Fig. 6C; Supplemental Fig. S6B). Loss of *nhr-19* but not *nhr-91* also blocked the enhanced learning capacity of *nkat-1* mutants (Supplemental Fig. S6C). While a role for *nhr-19* could not be ruled out, these results suggested that *nhr-91* is a better candidate for functioning between ADIOL and KYNA deficiency. Consistent with this, the learning-enhancing effects of WAY-200070 and F17 treatments were also lost in *nhr-91* mutants (Fig. 6C). Moreover, ADIOL-induced GCaMP activity of RIM neurons was abrogated in *nhr-91* mutants (Supplemental Fig. S6D). These studies suggest that NHR-91 functions analogous to mammalian ER-β. Based on DNA binding domain sequence homology, *nhr-91* has also been considered as a homolog of mammalian germ cell nuclear factor (Gissendanner et al. 2004; Kasuga et al. 2013). In that context, down-regulation of *nhr-91* mRNA is required to halt epidermal seam cell divisions during early larval stage (L1) diapause in response to starvation. Under ad libitum feeding conditions, ADIOL treatment did not cause gross changes to animal growth or development (data not shown).

To determine the sites of function of *nhr-91,* we generated transgenic animals expressing a transgene encoding 10 kb of *nhr-91* genomic sequence upstream of the stop codon bicistronically fused to GFP. Confocal microscopy revealed the presence of the transgene in select *C. elegans* glial cells and in RIM and RIC interneurons based on its colocalization with the coinjection marker for these interneurons (Fig. 6D), in addition to its previously reported expression in the spermatheca and vulva (Gissendanner et al. 2004). To ascertain the functional significance of the observed RIM expression, we restored full-length, wild-type *nhr-91* to *nhr-91* mutants using the RIM-specific promoter from *cex-1* (Piggott et al. 2011). The RIM-expressed *nhr-91* restored ADIOL's ability to elevate pharyngeal pumping (Fig. 6E) and learning (Fig. 6F) to a level indistinguishable from wild-type animals. However, we note that while, compared with nontransgenic controls, ADIOL treatment consistently enhanced learning in *nhr-91* mutants in which *nhr-91* was specifically reconstituted in the RIM neurons, the enhancement of learning induced by ADIOL in the transgenic animals did not reach statistical significance compared with the DMSO treatment of these transgenics. One potential reason for this is that even in the absence of treatment with exogenous ADIOL, RIM-specific reconstitution of *nhr-91* resulted in a slight, albeit statistically insignificant, increase in learning compared with *nhr-91* mutants (Fig. 1F). Given that we were reliant on a *cex-1* promoter to achieve RIM-specific reconstitution of *nhr-91*, we speculate that this promoter expresses *nhr-91* more robustly in the RIM neurons than the endogenous *nhr-91* promoter, which may lead to elevated RIM-specific NHR-91 activity in the absence of exogenous ADIOL. Nevertheless, we cannot rule out the possibility that *nhr-91* may contribute to learning regulation from cells other than the RIM neurons. However, examining the data in its totality indicates that RIM-specific restoration of *nhr-91* in *nhr-91* mutants allows these mutants to achieve the same elevated level of learning in response to ADIOL as that seen in similarly treated wild-type animals.

## Discussion

Using a combination of genetics, pharmacology, neuronal activity, and analytical biochemistry, we have found that the steroid hormone ADIOL is a critical product of a peripheral transcriptional program that promotes learning capacity by reducing neural KYNA levels in young or aged *C. elegans* (Fig. 6G). Biosynthesis of this steroid hormone is initiated in the intestine by the transcription factor NHR-131 and stimulated by exposure to F17. ADIOL mediates its effects in the nervous system through NHR-91, a likely ER-β homolog that is expressed in kynurenic acid-producing RIM neurons. Intriguingly, ADIOL is effective in blunting the decline in learning capacity that is seen in aged animals. The efficacy of ADIOL in enhancing learning capacity is similar to that seen with caloric or dietary restriction (Vohra et al. 2017). However, unlike these treatments, ADIOL does not extend the life span of *C. elegans*. Thus, the learning-promoting effects of ADIOL are not simply secondary consequences of increased life span.

While the effects of the kynurenic acid on learning occur locally within the nervous system, the transcriptional program that ultimately generates ADIOL largely acts in the *C. elegans* intestine, a metabolically active peripheral tissue in this organism. Thus, ADIOL is well positioned to serve as an endocrine mechanism for linking peripheral metabolic pathways to neural mechanisms of learning and memory.

Our discovery of the *nhr-131* transcriptional circuit that led us to ADIOL was made possible by F17, a novel small molecule. We speculate that F17 mimics either an environmental or a physiological cue to which NHR-131 is responsive. However, the identities of such cues are currently unknown to us. One potential clue to mechanisms that may regulate ADIOL generation is that *cyp13A4*, which is required for ADIOL biosynthesis, was previously described as an insulin-regulated gene (Murphy et al. 2003). Similarly, the likely 17β-HSD encoded by *F12E12.11*, which is also likely required for ADIOL generation, is named *nta-1* because of an altered nictation phenotype, a nematode behavior that is pronounced in dauer larva and regulated by insulin and TGF-β signaling (Lee et al. 2017). Thus, developmental or nutritional cues that act through insulin or TGF-β signaling may participate in the regulation of ADIOL levels.

It has long been known that humans biosynthesize ADIOL (Adams 1985), but this steroid has been largely overlooked and typically thought to be a minor intermediate in the C19 steroid biosynthesis (Adams 1985; Miyamoto et al. 1998). Given the unambiguous conservation of most of the components, both metabolic and genomic, of the outlined pathway, we predict that ADIOL will also promote learning capacity in mammals.

Although activation of ER-β has been intensively studied because of its potential as an antitumor pathway (Mal et al. 2020), functions for this receptor in other organ systems such as the immune system (Chakraborty et al. 2022), cardiovascular system (Aryan et al. 2020), and nervous system (Weiser et al. 2008) have also been identified. Relevant to our findings, activation of ER-β has been shown to improve learning and memory in mammals (Taxier et al. 2020). The potential beneficial effects of ER-β activation have also been noted as being neuroprotective in models of brain injury (Chakrabarti et al. 2014; Guo et al. 2020) and in improving mitochondrial function in the presence of soluble amyloid oligomers (Sarkar et al. 2015), as well as having an anxiolytic effect (Hughes et al. 2008). Conversely, ER-β knockout mice display deficits in learning behavior (Rissman et al. 2002; Day et al. 2005). Despite the emerging evidence of the effects of ER-β on learning and memory, the mechanisms that underlie these beneficial effects have remained poorly understood. Our findings suggest that reductions in KYNA likely account for the beneficial effects of ER-β activation on learning and memory. Notably, KYNA levels are known to affect learning and memory in mammals (Chess and Bucci 2006; Chess et al. 2007; Potter et al. 2010; Pocivavsek et al. 2011; Alexander et al. 2012; Kozak et al. 2014). Although increases in KYNA have been thought to provide protection in conditions characterized by glutamate toxicity, reductions in KYNA have also been considered as a possible therapeutic strategy in some CNS disorders (Savitz 2020). ADIOL/ER-β signaling represents a mechanism by which levels of KYNA may be controlled in a dynamic and potentially spatially restricted manner within the brain to improve cognitive functions.

## Materials and methods

### Data visualization and statistics

All graphically represented data were generated using the packages dplyr, drc, reshape2, and ggplot2 in R (https://ggplot2.tidyverse.org; https://CRAN.R-project.org/package=dplyr; https://www.R-project.org; Wickham 2007; Ritz et al. 2015). Unless indicated otherwise, feeding data are presented as box plots bounding the 25th, 50th, and 75th percentiles and whiskers terminating at the 5th and 95th percentile. Individual learning trials are plotted along with summary statistics represented by crossbars denoting the mean, upper, and lower 95% confidence intervals for each condition. Metabolite data from independent biological samples are plotted along with summary statistics bar plots representing the mean and 95% confidence interval with error bars. GCaMP data are presented as a heat map of stacked individual recordings, and the summed intensities of each recording are plotted adjacently on top of summary box and whiskers plots indicating the same percentiles as with the feeding data. RNA-seq data are presented as row-normalized means in heat maps and as binned histograms representing the number of genes at each level of induction or repression. Feeding and learning data were analyzed by either one- or two-way ANOVA, depending on the number of experimental variables. Post-hoc significance testing of one-way ANOVA was performed using Tukey's honestly significant difference test. Post-hoc testing of groups containing the significant variable from two-way ANOVA used *t-*tests corrected for multiple comparisons using Holm's method. In the case of dose–response data, Dunnett's test was used to correct for multiple comparisons of each dose versus vehicle control data. Metabolite data were evaluated using a *t*-test unless more than two groups were involved, in which case ANOVA was performed with post-hoc testing using Holm's correction for multiple comparisons. Integrated GCaMP response data were evaluated using either Wilcoxon rank-sum or Kruskal–Wallis test followed by Dunn's posttest. The significance of gene expression changes in the RNA-seq data was measured using the Wald test *P*-value and corrected for a 5% false discovery rate using the method of Benjamini–Hochberg (Benjamini and Hochberg 1995). In addition to R base packages, the following packages were used to statistically evaluate the data: rstatix, car, DescTools, DESeq2, and NbClust (https://CRAN.R-project.org/package=rstatix; https://socialsciences.mcmaster.ca/jfox/Books/Companion; Charrad et al. 2014; Love et al. 2014).

### Strains

Strains used in this study are listed in Supplemental Table S1. Unless noted otherwise, *C. elegans* were cultured at 20°C on *Escherichia coli* OP50 on NGM agar plates as described (Brenner 1974). Mutant alleles were backcrossed four generations into the wild-type N2 strain of the laboratory. Transgenic strains were made by injecting plasmid DNA into the gonad arm.

### Chemicals

Chemicals used in this study were as follows: F17 (custom synthesis, TimTec); 5-androstene 3β, 17β-diol; testosterone (Steraloids); dehydroepiandrosterone; pregnenolone; progesterone; butanone; DHEA-2,2,3,4,4,6-D_6_; pregnenolone-20,21-^13^C_2_-16,16-D_2_ (Millipore-Sigma); 5-androstene 3β,17β-diol-16,16,17-D_3_ (Cambridge Isotope Laboratories); 17-β estradiol; WAY-200070; and DPN (Tocris). All solvents used in extraction, derivatization, and chromatography were HPLC-grade or higher. S-basal was comprised of 100 mM NaCl and 25 mM potassium phosphate (pH 6), and S-medium was made using S-basal, 5 μM cholesterol, 10 mM potassium citrate (pH 6) trace metals, 3 mM CaCl_2_, and 3 mM MgCl_2_.

### General molecular biology

Plasmids used to make transgenic extrachromosomal arrays were prepared by standard PCR-based amplification of *C. elegans* genomic DNA or cDNA using either the Gateway system (Thermo Fisher Scientific) or NEBuilder Hi-Fi DNA assembly (New England Biolabs) recombinational cloning techniques. The fragments of genomic DNA used for the promoters in this study were as follows (kilobases 5′ to the ATG start site): *nhr-131*: 1.1 kb, *cex-1*: 0.9 kb, *c*y*p-13A4*: 0.5 kb, *F12E12.11*: 0.7 kb, *nhr-131*: 1.1 kb, *nhr-91*: 5.3 kb, *nmr-1*: 1.1 kb, *tdc-1*: 2.1 kb, and *vha-6*: 0.9 kb. For the *lim-7* promoter, 1.8 kb of sequence spanning the first intron was used. Both *nhr-131* and *nhr-91* reporter constructs included the complete genomic coding sequence followed by a bicistronic GFP (sl2::GFP). The 3′ untranslated sequence of the *unc-54* gene was used for all constructs. *F12E12.11* (0.85-kb) and *nape-2* (0.76-kb) RNAi constructs were cloned by PCR amplification of cDNA and ligated into Not1-digested pL4440. A neon green (nGreen) cassette was engineered into the genome before the stop codon of *cyp-13A4* using a CRISPR method (Ghanta and Mello 2020) to enable an endogenous C-terminal translational fusion to be produced. A deletion allele in *F12E12.11* was created by CRISPR mutagenesis (Paix et al. 2015), resulting in a 640-bp deletion of the genome. The sequences flanking the deletion in the genomic coding sequence were 5′: CAACAAGGAGCAAAGGTAACC (exon2) and 3′: TTCCGAAAATTGCTCCTGTTT (intron2).

### Pharmacological treatments

Unless indicated otherwise, *C. elegans* were treated with 2.5 μM F17, 50 nM pregnenolone, 15 nM DHEA, 10 nM ADIOL, 200 nM WAY-200070, or 0.1% DMSO as a vehicle control. NGM or RNAi plates containing compounds or vehicle alone were prepared 1 d prior to the commencement of treatment by diluting the compound in a suspension of *E. coli* used to seed the plate to create a lawn the next day. *C. elegans* used in behavioral assays or spontaneous Ca^2+^ assays were added to the plates as L4 animals, cultured overnight, and then assayed as day 1 adults unless described otherwise.

### Pharyngeal pumping

Pharyngeal pumping was assayed as described in day 1 gravid adults unless described otherwise (Lemieux et al. 2015). To understand the kinetics of F17-stimulated pharyngeal pumping, day 1 adults raised without F17 were transferred using a pick to either F17-containing plates or vehicle control plates, and the pharyngeal pumping rates were quantified 1, 2, 3, and 4 h after transfer.

### Short-term associative learning

Short-term associative learning was performed as described (Vohra et al. 2017) with minor modifications. Day 1 adult animals were conditioned with butanone in ethanol by streaking 3 μL of a 10% (v/v) solution in ethanol on the underside of the lid on a 6-cm plate and then incubated in a humidified chamber for 35–50 min at 20°C. Conditioning was terminated by the removal of the plate lid, and *C. elegans* were washed from the conditioning plate in 1 mL of S-basal + 0.05% PEG-8000 and transferred to 1.5-mL tubes to settle for ∼1–2 min. The settled pellet was aspirated in <50 μL and transferred to the origin of a 10-cm chemotaxis plate (Vohra et al. 2017). Excess moisture was absorbed using a folded tissue to release animals for chemotaxis. After 1 h of chemotaxis at 20°C, chemotaxis plates were transferred to a 4°C cold room to arrest movement until plates could be scored. The chemotaxis index (CI) = (animal count on butanone − animal count on ethanol)/(total animals on the plate). Animals that did not migrate away from the origin were excluded from the analysis. The learning index was the difference in CI between butanone-conditioned animals and the CI of naive animals.

### KYNA determination

Liquid cultures were grown on a shaker at 150 rpm in a 125-mL Erlenmeyer flask containing initially 25,000 L1 larvae in a 25-mL *E. coli* OP50 suspension (OD 600 nm ∼4) in S-medium. The L1 larvae were grown overnight, treated with 5 mL of an OP50 suspension containing either 0.6% DMSO or 15 μM F17, and continued to be shaken overnight. L4 cultures were harvested by centrifugation, washed with S-basal once, purified by flotation on 30% sucrose at 4°C, and washed once with cold 100 mM NaCl and 10 mM potassium citrate (pH 6), and pellets were frozen at −80°C until processing. To determine KYNA, 200 μL of water and 0.5-mm ZrO beads was added to each pellet, and the samples were lysed by bead beating at 4°C (six cycles of 20 sec on and 60 sec off). The homogenate was isolated by aspiration from the beads and then clarified by centrifugation at 16,500*g* for 10 min. A portion of the clarified supernatant was assayed for protein content using a Lowry assay (Bio-Rad), and 150 μL of supernatant was acidified using 50% trichloroacetic acid to precipitate protein. The acidified mixture was then clarified by centrifugation and assayed as described (Lemieux et al. 2015) by C_18_ reverse-phase HPLC (100-μL injection on a 4.6 × 100-mm column) elution with an isocratic mobile phase (4% acetonitrile, 250 mM Zn acetate at pH 6.2) at 1.0 mL/min interfaced with a fluorescence detector (350-nm excitation and 415-nm detection). The AUC for KYNA was converted to a concentration using a standard curve of KYNA concentrations chromatographed under identical conditions and normalized to the protein content of the sample.

### qRT-PCR

RNA from 300 L4 *C. elegans* treated for 4 h with either DMSO or F17 was extracted with Trizol, isolated, DNase-treated using a miniprep kit (Zymo Research), and converted to cDNA (NEB first strand synthesis, New England Biolabs) using random primers. SYBR Green-based qPCR was performed using a master mix from KAPA Biosystems. The gene-specific primer sequences used were as follows: *aat-1*-F (TGGATTGAGGCTATCGTAGTC), *aat-1*-R (TGCCAACCGAACAGAAATAC), *nkat-1*-F (GTTCCATGTATCTCAGCCG), *nkat-1*-R (ATTGCCCATCCGAGTTTC), *kmo-1*-F (CGTCGAAGATACCTACTTTTGG), *kmo-1*-R (TGGGAAAAGTCAAATCTCGG), *tdo-2*-F (AGTCTTCAGTTCCGTGTATTG), *tdo-2*-R (TCAATCCTGGTGTTCTTTCC), *nkat-3*-F (GCGGATGAGGTTTATGAGTTC), *nkat-3*-R (AGTTCTGGTGAATGGCTTTC), *act-1*-F (CACCATGTACCCAGGAATTG), *act-1*-R (TGTTGGAAGGTGGAGAGG), *tba-1*-F (ACACTCCACTGATCTCTGC), and *tba-1*-R (CAGCCATGTACTTTCCGTG). The geometric mean *C*_*t*_ of the responses for the *act-1* and *tba-1* genes was used to normalize for sample variance in total cDNA abundance.

### Forward mutagenesis screening

Synchronized L4 larvae were mutagenized by exposure to ethyl methane sulfonate (EMS) for 4 h at room temperature (Brenner 1974). The animals were washed free of EMS and then plated on OP50/NGM plates to develop into egg-laying adults. F1 progeny were obtained by sodium hypochlorite treatment of gravid P0 adults and synchronized as L1 larvae by hatching overnight in the absence of food. Forty-eight aliquots of 250 F1 progeny were cultured for 3 d at room temperature on 6-cm NGM plates, and then eggs were harvested by alkaline hypochlorite treatment. The F2 progeny from each F1 aliquot were synchronized by hatching in the absence of food, and then 5000 animals from each culture were grown in S-medium supplemented with OP50, 100 nM Nile Red, and 2.5 μM F17. The cultures were grown for 2 d and then plated on 10-cm NGM plates containing a lawn of OP50. The plates were examined using an epifluorescence stereomicroscope for rare animals that exhibited strong red intestinal fluorescence. Positive animals were backcrossed four generations into the nonmutagenized wild-type background, using F17 and Nile Red to screen cross-progeny for F17 resistance mutations.

### Mutant sequencing

Genomic DNA from each suppressor line and the nonmutagenized wild-type strain were extracted using Proteinase K digestion, followed by purification using RNase digestion, phenol-chloroform extraction, and ethanol precipitation. The high-molecular-weight DNA was then subjected to focused ultrasonic shearing (Covaris) to generate <500-bp fragments. Barcoded adapter sequences were then added to the fragments (Nextflex PCR-free DNA-seq, Bioo Scientific/Perkin-Elmer) for each sample. Genomic libraries were quantified by PCR (NEBNext, New England Biolabs), pooled, and sequenced using paired-end 100-bp reads on an Illumina HiSeq3000. Sequencing data were analyzed using a pipeline described previously (Minevich et al. 2012) based in the Galaxy platform using the Burrows–Wheeler aligner to map the paired-end reads to the WBcel235 reference genome. The sequence obtained from the nonmutagenized wild-type strain was used to subtract variants from consideration for each suppressor line. Variants unique to the suppressor lines were functionally identified using SnpEff (Cingolani et al. 2012). The alleles affecting *nhr-131* and their predicted effect were identified as (1) c.55G>A: Gly19Arg, (2) c.70G>A: Val24Met, (3) c.227-2A>G: exon3 splice acceptor, (4) c.640-1G>A: exon4 splice acceptor, and (5) c.801G>A: Trp261STOP.

### Nile Red staining/epifluorescence microscopy

Starvation-synchronized L1 larvae were cultured for 2 d on plates containing 50 nM Nile Red and then transferred to plates containing 50 nM Nile Red and either 0.1% DMSO or 2.5 μM F17. Animals were anaesthetized with NaN_3_ and then imaged by epifluorescence microscopy using a 10× objective and red fluorescence excitation and emission filters. The integrated intensity from the first pair of intestinal cells was measured using ImageJ. Transgenic animals bearing fluorescent protein reporters were also anaesthetized with NaN_3_ and imaged using a 16× objective. DIC settings were used to obtain bright-field images, and GFP- and mCherry-compatible filter sets were used to image fluorescent protein expression.

### Ca^2+^ imaging

*C. elegans* were conditioned with butanone as described for “Short-Term Associative Learning,” mounted on 8% agarose pads in a drop of 0.1 μM polystyrene beads (Polysciences, Inc.), overlaid with a coverslip, and imaged using a GFP filter set, a 40× objective, 2 × 2-pixel binning, and a frame rate of 2 sec^−1^ for 250 sec. The first 25 sec of frames were omitted from the analysis to eliminate the contribution of the start of imaging to the neuronal transients. Images of the GCaMP3.0 intensities were measured using ImageJ. The integrated intensity from a region of interest defined by a 10-pixel-diameter circle centered on the GCaMP signal was acquired for each frame. The baseline fluorescence (*F*^*b*^) was obtained from the average of the 10th percentile image intensities in each time series. The difference in fluorescence intensity for each image from *F*^*b*^ (Δ*F*) was normalized to *F*^*b*^ (Δ*F/F*) and plotted versus time. Total integrated intensity is the sum of Δ*F/F* over each time series.

### Spontaneous movement

*C. elegans* strains were washed free of bacteria in S-basal and then transferred to unseeded NGM plates, and the number of body movements (sinusoidal oscillations, spontaneous reversals, and ω turns) were recorded over 30-sec intervals by direct observation using a stereomicroscope.

### Benzaldehyde chemotaxis

Six-centimeter NGM plates were divided into equal-sized quadrants using perpendicular drawn lines. On the edge of the plate, centered in each quadrant, 1.5 μL of 2% NaN_3_ was spotted, followed by alternating between 1.5 μL of 0.1% benzaldehyde in ethanol and ethanol alone in adjacent quadrants. Approximately 75–125 animals were transferred to the center of the plate in <30 μL of S-basal medium. The excess medium was removed by blotting with absorbent paper, and the animals were allowed to freely move for 1 h. The number of animals in benzaldehyde- and ethanol-containing quadrants was counted using a stereomicroscope; the few animals that did not move beyond 1 cm from the origin were not included in the analysis. The benzaldehyde chemotaxis index was calculated as (the number of benzaldehyde animals − the number of ethanol animals)/(the total number of animals).

### RNA-seq

Early L4 WT and *nhr-131* mutants in liquid culture (25,000 animals per culture, five biologically independent cultures per condition) were treated with either 0.1% DMSO or 2.5 μM F17 for 4 h. Animals were harvested by centrifugation, washed once with cold S-basal, cleaned by flotation on ice-cold 30% sucrose, and then washed once with ice-cold S-basal. Trizol was added to the pellets, and the samples were flash-frozen in liquid N_2_ and then stored at −80°C. Total RNA was extracted and isolated using spin columns coupled with on-column DNase digestion (Zymo Research). Total RNA (1 μg/sample) was converted into indexed Illumina adapter-ligated cDNA libraries using polyA magnetic beads [Nextflex poly(A) beads, Bioo Scientific/Perkin Elmer] in conjunction with a library synthesis kit (NextFlex rapid directional RNA-seq kit, Bioo Scientific/Perkin Elmer). Libraries were quantified by PCR (NEBNext, New England Biolabs), pooled equally, and sequenced on an Illumina HiSeq4000 using paired-end 100-bp reads. In the Galaxy platform, reads were mapped to the WBcel235 genome using TopHat2 (Kim et al. 2013), and raw counts were obtained using HTSeq-count (Anders et al. 2015). Differential expression analysis of the raw count matrix was performed using the package DESeq2. To quantify expression differences, at least 50 mapped reads across the 20 experimental groups were required for a gene to be included in the analysis. Contrasts generated included genes significantly changed at a Benjamini–Hochberg-adjusted false discovery rate of ≤5% in F17-treated versus DMSO-treated wild-type animals (F17-regulated genes) and in DMSO-treated *nhr-131* mutants versus DMSO-treated wild-type animals (*nhr-131*-regulated genes), as well as genes that F17 differentially regulates in wild type versus *nhr-131* mutants (F17-regulated, *nhr-131*-dependent genes). The number of clusters that represented >90% of the variance in gene expression across both the genotypes and treatments was found to be three using the NbClust package in R. *k-*means clustering based on three classes was used to sort genes into each class.

### RNAi

For feeding screens, *E. coli* HT115-containing RNAi clones from the Ahringer (Kamath et al. 2003) or Vidal (Rual et al. 2004) genome-wide libraries or empty L4440 as a vector control were inoculated into LB supplemented with ampicillin and cultured overnight at 37°C. Double-stranded RNA production was induced by IPTG for 2 h at 37°C and then plated onto NGM plates containing 100 μg/mL carbenicillin and IPTG overnight. Synchronized L1 animals were transferred to the plates and cultured for 2 d, transferred to plates containing the identical induced RNAi clone supplemented with either compounds or vehicle control, and cultured overnight.

### Life span

A timed egg-lay (4 h) was used to create cultures of age-synchronized *C. elegans* (45–55 animals per plate) that were cultured on OP50 lawns from hatching containing either ADIOL or DMSO at 20°C. After 2.5 d of culture, animals were transferred to OP50-seeded plates containing 120 μM FUDR (5-fluoro-2-deoxyuridine) and either ADIOL or DMSO. After 5 d of culture, animals were transferred again to OP50-seeded plates lacking FUDR but containing either ADIOL or DMSO. Cultures were examined three times per week for dead adults until no surviving adults were present. Dead animals were defined as those that did not move in response to a light touch on the head.

### ADIOL determination

Liquid cultures of 200,000 L1 animals were grown on a shaker at 150 rpm for 30 h and then treated with either 0.1% DMSO or 2.5 μM F17 for an additional 12 h until animals were mid-L4. The cultures of animals were pelleted by centrifugation, washed once with cold S-basal medium, purified by flotation on ice-cold 30% sucrose, washed once with S-basal, snap-frozen in liquid nitrogen, and stored at −80°C. Frozen pellets were homogenized in water by bead-beating (see “KYNA Determination”). After aspiration of the homogenate from the beads, the homogenates were acidified using 1 N HCl.

#### Sample extraction

*C. elegans* homogenates (0.4 mL of 133,000 L4 stage animals/mL in 0.1 M HCl) were spiked with 5 μL of a solution of isotope-labeled internal standards (5 μM ADIOL-D_3_, 5 μM DHEA-D_6_, 5 μM pregnenolone-C^13^_2_D_2_) in methanol. For standard spike control samples, 5 μL of 10 uM standard solution (pregnenolone, DHEA, and ADIOL) was added to homogenate from wild-type animals. The homogenates were extracted with 2 mL of methyl tertiary-butyl ether for 1 h at room temperature and centrifuged at 4000 rpm for 5 min. The clarified upper phase was evaporated under a stream of N_2_, redissolved in 1-chlorobutane, and applied to a solid-phase extraction cartridge bed (100 mg/mL SiO_2_, 55 μm, and 70 Å; Strata SI-1 Silica). The bed was washed with hexanes and 5% ethyl acetate in hexanes, eluted with 50% ethyl acetate in hexanes, and evaporated to dryness under N_2_.

#### Nicotinate derivatization

Fifty microliters of a solution of 20 mg/mL 1-ethyl-3-[3-dimethylaminopropyl]carbodiimide hydrochloride and 50 μL of a 1:1 solution of 54 mg/mL nicotinic acid:4-dimethylaminopyridine in chloroform were added to the dried extracts and heated for 75 min to 65°C. The reaction was evaporated to dryness under a stream of N_2_, and the residue was reconstituted in 100 μL of 50% methanol for immediate analysis.

#### MS analysis

Nicotinate-derivatized samples were analyzed by LC-MS/MS using a Waters UPLC-XEVO-TQ-XS triple-quadrupole MS system operating in positive ionization, multiple reaction monitoring mode. Samples (5 μL/injection) were separated on an Acquity BEH C18 reverse-phase column (100 mm × 2.1 mm, 1.7 μm, 130 Å pore size) using a gradient elution protocol with mobile phase A (0.2% aqueous formic acid) and mobile phase B (0.2% formic acid in acetonitrile) at a flow rate of 0.1 mL/min over 12 min. The UPLC gradient was as follows: 40% B: 0–2 min, 40%–75% B: 2–9 min, 75%–100% B: 9–10 min, 100% B: 10–12.3 min, 100%–40% B: 12.3–13 min, and 40% B: 13–15 min. Mass spectrometer settings were as follows: capillary voltage: 3.0 kV, desolvation temperature: 500°C, desolvation gas (N_2_) flow: 1000 L/h, sheath gas (N_2_) flow: 150 L/h, and collision gas (Ar) flow: 0.16 mL/min. The cone voltage and collision energy were compound-dependent and, together with the mass transitions acquired, are shown in Supplemental Table S2.

#### Quantification

Peak areas from total ion chromatograms using two top mass transitions per steroid were integrated using Masslynx software and used for metabolite quantification. Peak areas from standards and samples were normalized to peak areas from the isotope-labeled internal standard most similar in elution time. Standard curves relating normalized peak areas to steroid concentration were linear over the quantification range used to quantify endogenous steroids. For each steroid, the lower limit of quantification at a signal to noise ratio of >10 was determined to be 0.1 fmol of ADIOL, 10 fmol of DHEA, and 0.5 fmol of pregnenolone on column.

### Confocal microscopy

*C. elegans* were anesthetized with 3 μL of 200 mM sodium azide and imaged on 2% agarose pads under a glass coverslip using a Nikon Ti2-E microscope equipped with a Crest X-Light V2 L-FOV spinning-disk confocal, MCL piezo stage, Prime 95B 25-mm sCMOS camera. Excitation lines and emission filters were 477 nm and 511/20 nm for GFP and 546 nm and 595/31 nm for mCherry. Images were collected using a 60×/1.4 NA objective.

### Data and material availability

Raw read data from RNA-seq have been deposited at GEO (GSE221221). All other data are available here or in the Supplemental Material.

## Supplementary Material

Supplement 1

Supplement 2
