# Supplementary material for: The steroid hormone ADIOL promotes learning by reducing neural kynurenic acid levels
Source: Genes Dev. 2023 Nov-Dec;37(21-24):998–1016. doi: 10.1101/gad.350745.123 (PMC10760639; doi:10.1101/gad.350745.123)
Supplement: Supplement 2 [file Supplemental_Figures_Tables.pdf]

Supplemental Figure S1

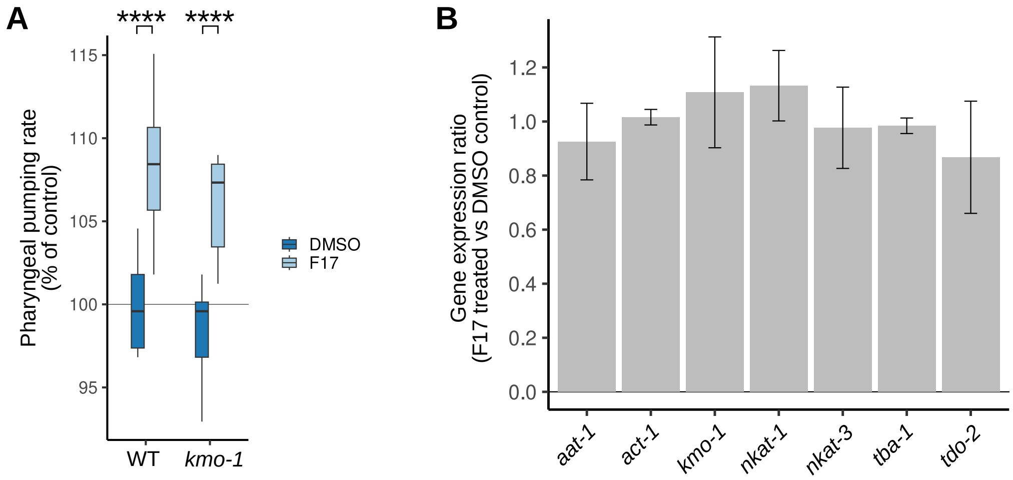

Figure S1. Gene expression and the influence of extended F17 treatment on feeding. (**A**) Pharyngeal pumping rates of L4 animals following 2 days of treatment with F17 or DMSO. n = 16 animals per condition **** $p < 0.0001$ ANOVA (Holm's correction). (**B**) The ratio of the expression of each gene in F17 vs DMSO treated animals. *aat-1*, *kmo-1*, *nkat-1*, *nkat-3*, *tdo-2* are kynurenine pathway genes. *act-1* and *tba-1* are normalization standards. Error bars represent the standard error of the mean, n = 3 independent biological samples per condition. None of the genes exhibit a statistically significant change in expression by ANOVA.

Supplemental Figure S2

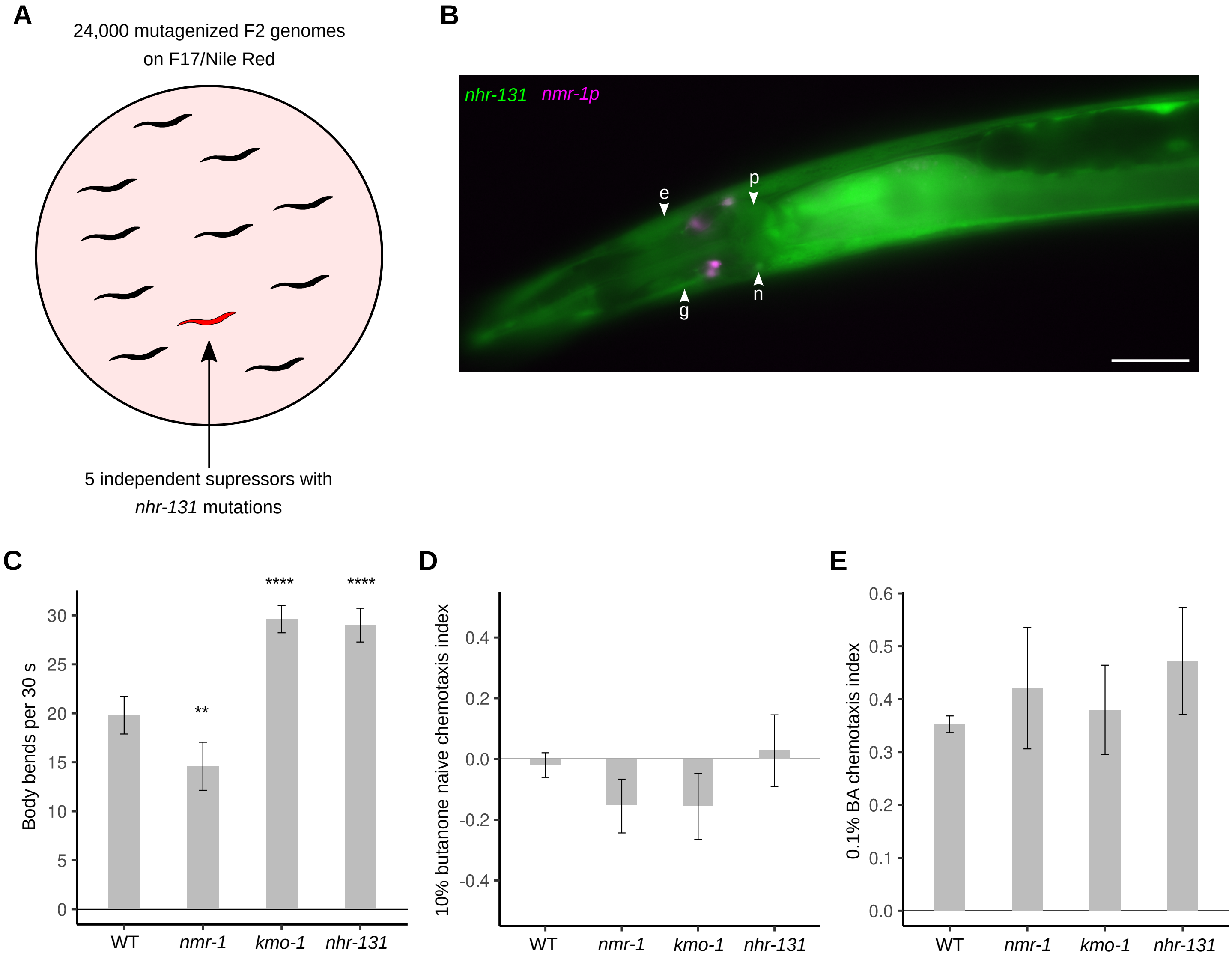

Figure S2. Mutagenesis screen strategy, higher magnification image of a *nhr-131p::GFP* transgenic, and parameters relevant to the learning assay. (**A**) 24,000 mutagenized genomes were examined in the F2 generation for their ability to suppress the Nile Red reducing effects of F17. (**B**) Composite fluorescence micrograph of the *nhr-131p::nhr-131bcsGFP*, *nmr-1p::mCherr*y transgenic animal shown in Figure 2 at a higher magnification and different focal plane. Although very weak relative to intestinal and somatic gonad expressions, the zoomed-in image shows *nhr-131p::nhr-131bcsGFP* expression in other cell types. Epidermis (e), glia (g), neuron (n), pharyngeal muscle (p). Scale bar = 30 μm. (**C**) Spontaneous movement of wild type (WT), *nmr-1*, *kmo-1*, and *nhr-131* mutants. n = 15 animals per condition. Error bars represent the 95% confidence interval. ANOVA(Holm's correction. ** p<0.01, ****p<0.0001. (**D**) Naive chemotaxis indices of wild type, *nmr-1*, *kmo-1*, and *nhr-131* mutants to 10% butanone. n = 12-68 trials per condition. Error bars represent the 95% confidence interval. No signficant differences at a $p < 0.05$ ANOVA(Holm's correction) (**E**) Chemotaxis ability of wild type, *nmr-1*, *kmo-1*, and *nhr-131* mutants to 0.1% benzaldehyde (BA). n = 4 independent trials per genotype, error bars represent the standard error of the mean.

Supplemental Figure S3

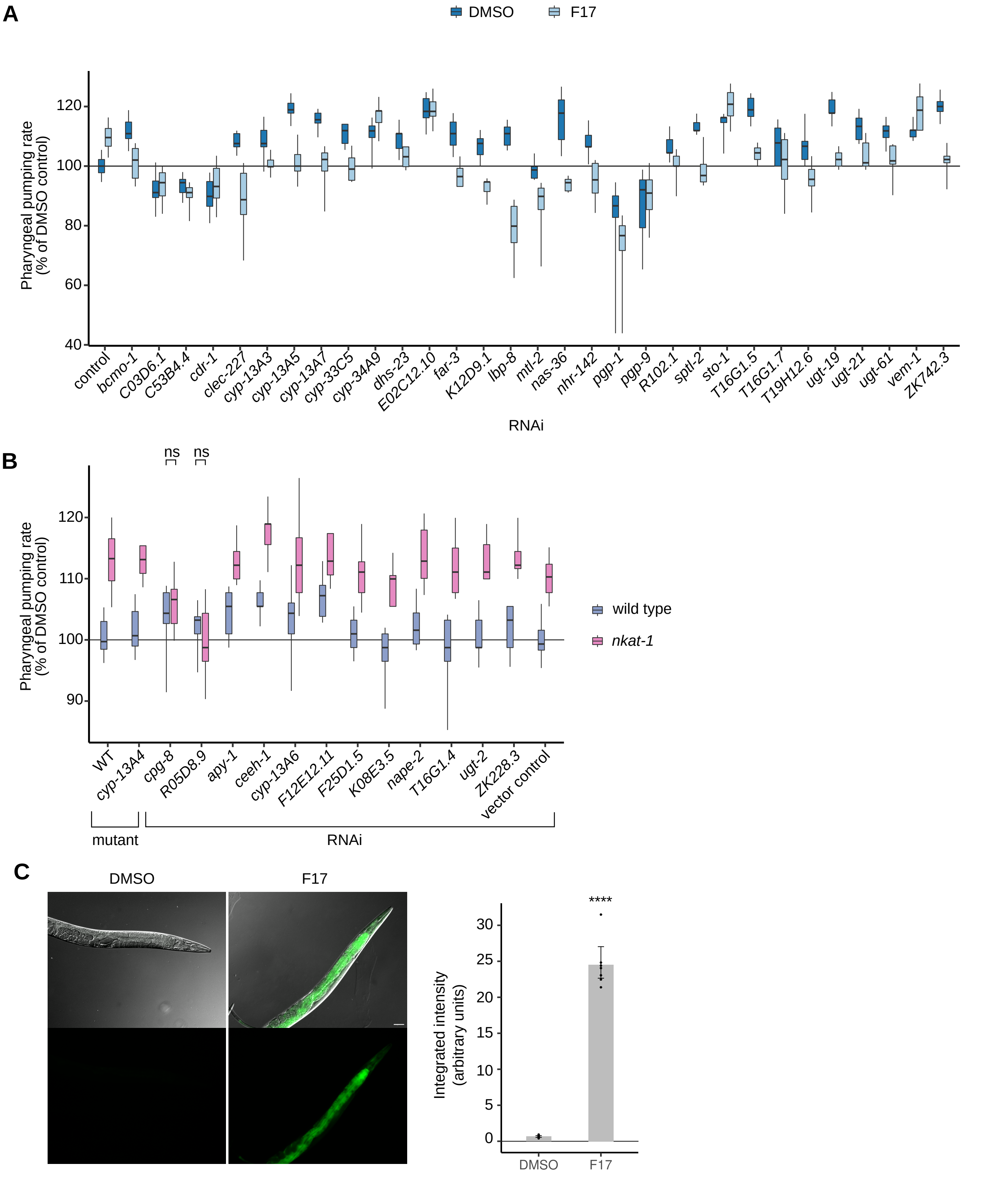

Figure S3. Effects of a subset of F17 regulated genes on pharyngeal pumping rate and expression pattern of endogenously tagged *cyp13A4*. (**A**) Pharyngeal pumping in animals treated with either DMSO or F17 and subjected to the indicated RNAi. All RNAi treatments alter the pumping rates of F17-treated animals compared to F17-treated vector control ($p < 0.05$). In some cases, this was due to the RNAi's effects on baseline pumping rates in the absence of F17 (DMSO group). Statistical evaluation by ANOVA (Holm's correction). n = 10-130 per group. The vector control groups are the same population as that used in Figure 3A. (**B**) The pharyngeal pumping rates of *nkat-1* mutants subjected to the subset of RNAis that blocked the pumping increasing effects of F17 without altering baseline levels of pumping rate (same RNAis as those shown in Figure 3A). For comparison, the effects of *nkat-1* RNAi on wild type or *cyp13A4* mutants are shown. Except for pairs of interactions denoted ns ($p > 0.05$), wild type animals exhibited significantly different pharyngeal pumping rates ($p < 0.01$ Holm multiple comparisons adjusted *t*-test) from animals with inactivated *nkat-1* in each genetic background. (**C**) CYP13A4::nGREEN expression pattern in animals bearing a *nGreen* cassette integrated into the *cyp13A4* genomic locus. Composite micrographs of DIC and epifluorescence (*top panels*) and epifluorescence alone (*bottom panels*) after both DMSO and F17 treatment are shown. Scale bar = 30 µm. The integrated fluorescence intensity in the intestine was quantified. n = 7 animals per condition, error bars represent the 95% confidence interval. **** $p < 0.0001$ (*t*-test).

Supplemental Figure S4

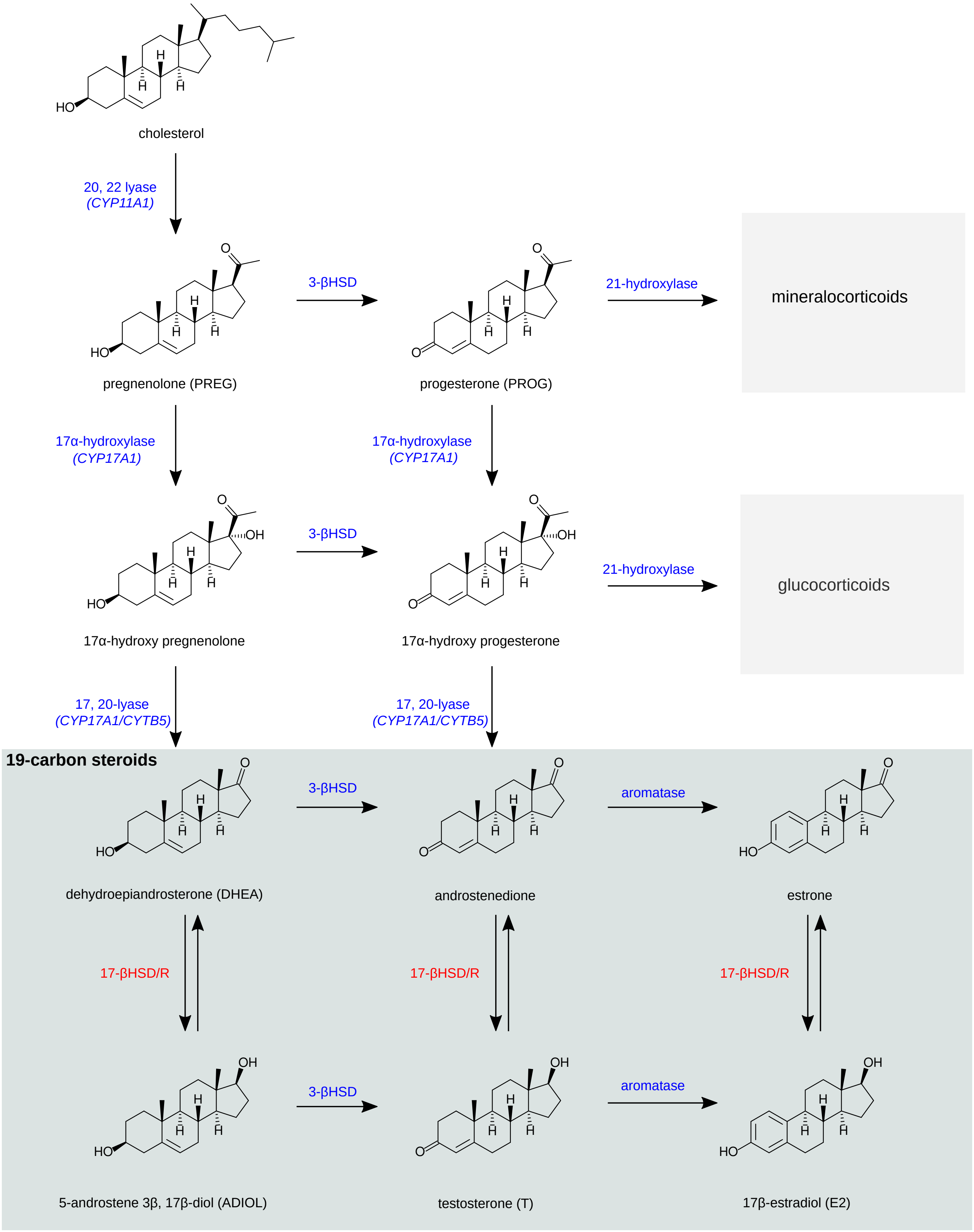

Figure S4. Metabolism of cholesterol into 19 carbon steroids and the enzyme activities involved.

Supplemental Figure S5

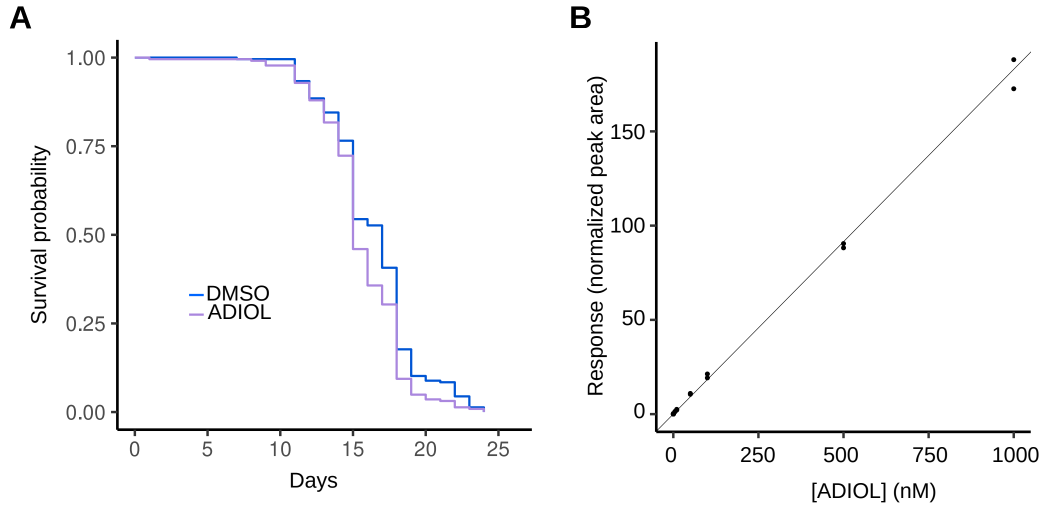

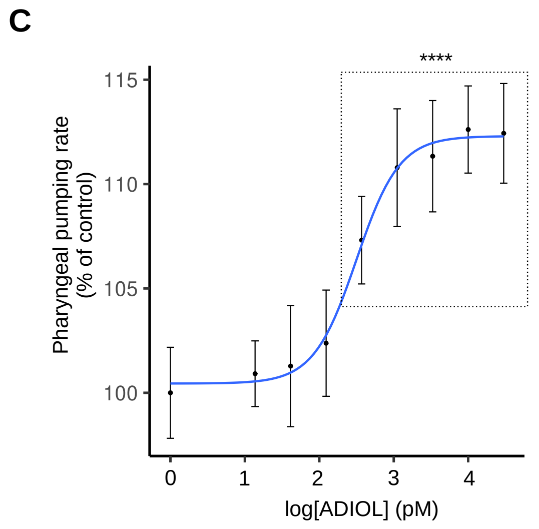

D

steroid	treatment	mean (nM)	s.e.m.
ADIOL	DMSO	0.33	0.07
ADIOL	F17	0.60	0.06
PREG	DMSO	0.83	0.12
PREG	F17	1.27	0.22

Figure S5. Experimental parameters relevant to steroid measurements. (**A**) Adult lifespans of animals treated with DMSO or ADIOL. Median survival time = 15 days (ADIOL), 17 days (DMSO). Cox proportional hazards ratio for ADIOL treatment = 1.3, p = 0.00114, n = 676 events. (**B**) ADIOL standard curve generated using the procedure outlined in Figure 5G. The peak areas for varying concentrations of ADIOL were normalized to those of a constant concentration of internal standard ADIOL-D_3. (**C**) Wild type animals were treated with different concentrations of ADIOL and pharyngeal pumping rates were measured. Points represent the mean of measurements from 12 different animals at each concentration of ADIOL and error bars represent the 95% confidence intervals of the mean. A four-parameter log-logistic model was used to generate the dose-response curve to estimate the EC_{50}. Dose points with measured means that are significantly different from the lowest vehicle dose are indicated: **** p < 0.0001 ANOVA (Dunnett's test) (**D**) ADIOL and PREG levels determined from extracts of DMSO and F17 treated animals. n = 3 independent biological samples per condition, s.e.m. (standard error of the mean).

Supplemental Figure S6

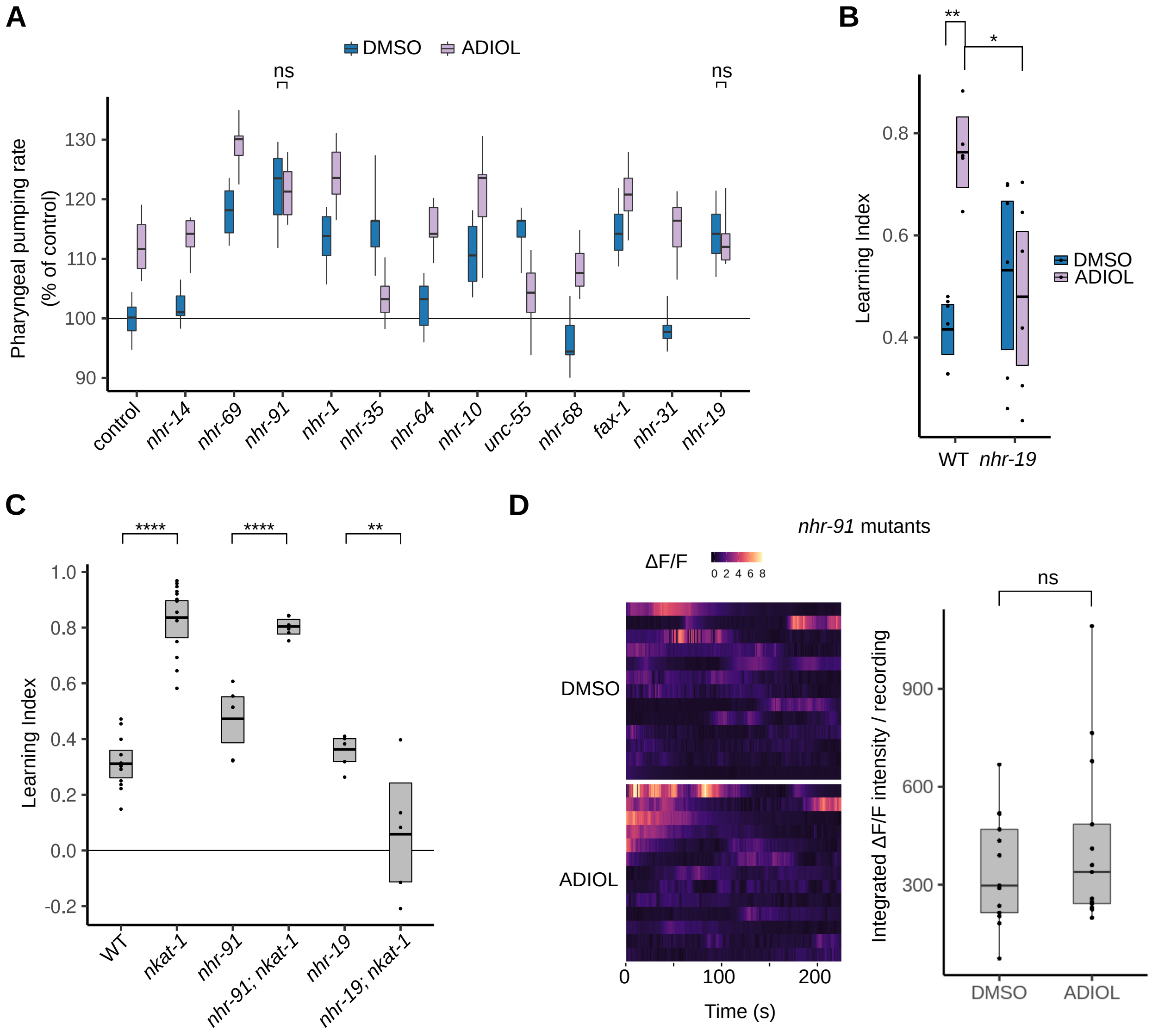

Figure S6. Characterization of a subset of nuclear hormone receptors with sequence homology to human estrogen receptor-β. (**A**) *C. elegans* were subjected to RNAi-mediated gene inactivations of the most orthologous sequences to human ER-β and treated with ADIOL or DMSO alone as a vehicle control. Pharyngeal pumping rates of 14-42 different animals per condition were measured. Statistical evaluation by ANOVA (Holm's correction). With the exception of interactions denoted as ns ($p > 0.05$) ADIOL significantly altered pharyngeal pumping in each case ($p < 0.01$). (**B**) Wild type and *nhr-19* mutants were treated with ADIOL or DMSO alone as a vehicle control, then assayed for learning. n = 5 to 6 trials per condition. ** $p < 0.01$, *$p < 0.05$ ANOVA (Holm's correction). (**C**) Wild type (WT) and the indicated mutants were assayed for learning. n = 5 to 11 trials per condition. **** $p < 0.0001$, ** $p < 0.01$ ANOVA (Tukey's HSD test). (**D**) Spontaneous GCaMP activity of RIM neurons in *nhr-91* mutants treated with either ADIOL or DMSO alone, then conditioned with butanone. 13 recordings of the change in fluorescence (ΔF/F) over 225 seconds (s) are shown per condition. The summed intensity of each recording is plotted. ns: $p = 0.5727$ Statistical evaluation Wilcoxon rank-sum test.

Supplemental Table S1. Strains used in this study

strain	genotype
KQ2668	*flp-18(gk3063)* X
KQ2669	*npr-5(ok1583)* V
KQ2670	*nmr-1(ak4)* II
KQ1072	*ser-5(tm2654)* I
KQ1194	*tph-1(mg280)* II
KQ1464	*nkat-1(ok566)* X
KQ2673	*kmo-1(tm4529)* V
KQ2674	*nhr-131(tm1376)* V
KQ2658	*nhr-131(tm1376)* V*; Ex[nhr-131p::nhr-131(genomic)::sl2::GFP, nmr-1p::mCherry]*
KQ2675	*nhr-131(tm1376)* V*; Ex[lim-7p::nhr-131(genomic)::sl2::GFP, tdc-1p::mCherry]*
KQ2676	*nhr-131(tm1376)* V*; Ex[cex-1p::nhr-131(genomic)::sl2::GFP, tdc-1p::mCherry]*
KQ2663	*nhr-131(tm1376)* V*; Ex[vha-6p::nhr-131(genomic), unc-122::GFP]*
KQ2677	*N2;Ex[cex-1p::GCaMP3, tdc-1p::mCherry]*
KQ2678	*nhr-131(tm1376)* V*; Ex[cex-1p::GCaMP3, tdc-1::mCherry]*
KQ2679	*N2; Ex[cyp-13A4p::GFP, odr-1p::RFP]*
KQ2680	*nhr-131(tm1376)* V; *Ex[cyp-13A4p::GFP, odr-1p::RFP]*
KQ2681	*cyp-13A4(ft1010[cyp-13A4::nGreen]) II*
KQ2682	*N2; Ex[F12E12.11p::GFP, odr-1p::RFP]*
KQ2683	*nhr-131(tm1376)* V*; Ex[F12E12.11p::GFP, odr-1p::RFP]*
KQ2684	*cyp-13A4(tm7443)* II
KQ2685	*F12E12.11(ft1005)* II
KQ2686	*nhr-19(tm886)* II
KQ2687	*nhr-91(tm4713)* X
KQ2688	*nhr-19(tm886)* II*; nkat-1(ok566)* X
KQ2689	*nhr-91(tm4713)* X*; nkat-1(ok566)* X
KQ2690	*nhr-91(tm4713)* X*; Ex[cex-1p::GCaMP3, tdc-1::mCherry]*
KQ2691	*nhr-91(tm4713)* X*; Ex[nhr-91p::nhr-91(genomic)::sl2::GFP, tdc-1p::mCherry, nmr-1p::mCherry]*
KQ2692	*nhr-91(tm4713)* X*; Ex[cex-1p::nhr-91a, tdc-1p::mCherry]*

Supplemental Table S2. Mass spectrometry settings used to measure each compound and internal standard.

Compound	Charge (+)	Precursor m/z	Fragment m/z	Quantified transition	Dwell time [sec]	Cone voltage [V]	Collision energy [eV]
ADIOL-bis-nicotinate	2	251.01	123.47		0.01	28	15
		251.01	250.31		0.01	28	56
		251.01	378.17	x	0.01	28	9
ADIOL-D_3-bis-nicotinate	2	252.54	123.66		0.01	30	15
		252.54	251.68		0.01	30	20
		252.54	381.14	x	0.01	30	9
DHEA-nicotinate	1	394.2	79.36		0.01	34	30
		394.2	105.48		0.01	34	26
		394.2	123.54	x	0.01	34	18
DHEA-D_6-nicotinate	1	400.21	77.38		0.01	34	30
		400.21	80.2		0.01	34	30
		400.21	96.44		0.01	34	30
		400.21	123.72	x	0.01	34	18
PREG-nicotinate	1	422.23	83.47		0.01	38	16
		422.23	123.53	x	0.01	38	22
		422.23	155.7		0.01	38	16
		422.23	299.82		0.01	38	16
PREG-$^{13}C_2D_2$-nicotinate	1	426.27	123.53	x	0.01	38	20
		426.27	310.97		0.01	38	20
